# Effects of colonization-associated gene *yqiC* on global transcriptome, cellular respiration, and oxidative stress in *Salmonella* Typhimurium

**DOI:** 10.1186/s12929-022-00885-0

**Published:** 2022-12-01

**Authors:** Hung-Hao Fan, Shiuh-Bin Fang, Yu-Chu Chang, Sheng-Tung Huang, Chih-Hung Huang, Pei-Ru Chang, Wei-Chiao Chang, Lauderdale Tsai-Ling Yang, Pei-Chun Lin, Hung-Yen Cheng

**Affiliations:** 1 Division of Pediatric Gastroenterology and Hepatology, Department of Pediatrics, Shuang Ho Hospital, Taipei Medical University, No. 291, Jhong Jheng Road, Jhong Ho, New Taipei City, 23561 Taiwan; 2 Department of Pediatrics, School of Medicine, College of Medicine, Taipei Medical University, Taipei, Taiwan; 3 Department of Emergency Medicine, Shuang Ho Hospital, Taipei Medical University, New Taipei City, Taiwan; 4 Master Program for Clinical Genomics and Proteomics, College of Pharmacy, Taipei Medical University, Taipei, Taiwan; 5 Department of Biochemistry and Molecular Cell Biology, School of Medicine, College of Medicine, Taipei Medical University, Taipei, Taiwan; 6 Graduate Institute of Biochemical and Biomedical Engineering, National Taipei University of Technology, Taipei, Taiwan; 7 National Institute of Infectious Diseases and Vaccinology, National Health Research Institutes, Zhunan, Taiwan

**Keywords:** *yqiC*, *Salmonella* Typhimurium, Global transcriptome, RNA sequencing (RNA-seq), Colonization, Electron transport chain (ETC), Glycolysis, Ubiquinone (UQ), Menaquinone (MK), Oxidative stress

## Abstract

**Background:**

*yqiC* is required for colonizing the *Salmonella enterica* serovar Typhimurium (*S*. Typhimurium) in human cells; however, how *yqiC* regulates nontyphoidal *Salmonella* (NTS) genes to influence bacteria–host interactions remains unclear.

**Methods:**

The global transcriptomes of *S*. Typhimurium *yqiC*-deleted mutant (Δ*yqiC*) and its wild-type strain SL1344 after 2 h of in vitro infection with Caco-2 cells were obtained through RNA sequencing to conduct comparisons and identify major *yqiC*-regulated genes, particularly those involved in *Salmonella* pathogenicity islands (SPIs), ubiquinone and menaquinone biosynthesis, electron transportation chains (ETCs), and carbohydrate/energy metabolism. A Seahorse XFp Analyzer and assays of NADH/NAD^+^ and H_2_O_2_ were used to compare oxygen consumption and extracellular acidification, glycolysis parameters, adenosine triphosphate (ATP) generation, NADH/NAD^+^ ratios, and H_2_O_2_ production between Δ*yqiC* and SL1344.

**Results:**

After *S*. Typhimurium interacts with Caco-2 cells, *yqiC* represses gene upregulation in aspartate carbamoyl transferase, type 1 fimbriae, and iron–sulfur assembly, and it is required for expressing *ilvB* operon, flagellin, *tdc*ABCD, and *dms*AB. Furthermore, *yqiC* is required for expressing mainly SPI-1 genes and specific SPI-4, SPI-5, and SPI-6 genes; however, it diversely regulates SPI-2 and SPI-3 gene expression. *yqiC* significantly contributes to *menD* expression in menaquinone biosynthesis. A Kyoto Encyclopedia of Genes and Genomes analysis revealed the extensive association of *yqiC* with carbohydrate and energy metabolism. *yqiC* contributes to ATP generation, and the analyzer results demonstrate that *yqiC* is required for maintaining cellular respiration and metabolic potential under energy stress and for achieving glycolysis, glycolytic capacity, and glycolytic reserve. *yqiC* is also required for expressing *ndh*, *cydA*, *nuoE*, and *sdhB* but suppresses *cyoC* upregulation in the ETC of aerobically and anaerobically grown *S*. Typhimurium; priming with Caco-2 cells caused a reversed regulation of *yiqC* toward upregulation in these ETC complex genes. Furthermore, *yqiC* is required for maintaining NADH/NAD^+^ redox status and H_2_O_2_ production.

**Conclusions:**

Specific unreported genes that were considerably regulated by the colonization-associated gene *yqiC* in NTS were identified, and the key role and tentative mechanisms of *yqiC* in the extensive modulation of virulence factors, SPIs, ubiquinone and menaquinone biosynthesis, ETCs, glycolysis, and oxidative stress were discovered.

**Supplementary Information:**

The online version contains supplementary material available at 10.1186/s12929-022-00885-0.

## Background

Nontyphoidal *Salmonella* (NTS) is a common foodborne enteropathogen found in humans and animals worldwide, and it contributes considerably to morbidity and mortality [1]. Bacterial colonization in the host intestinal epithelium is essential for the successful establishment of NTS infection [2]. In *Salmonella enterica* subsp. *enterica* serovar Typhimurium (*S*. Typhimurium), the YqiC encoded by *yqiC* was first reported to be responsible for bacterial survival involving thermosensitivity and for host survival in mice; however, *yqiC* depletion does not attenuate bacterial invasion and intracellular replication in J774 murine macrophages and human epithelial HeLa cells [3]. By contrast, through the transposon-directed insertion-site sequencing of 1440 transposon mutants of *S*. Typhimurium str. SL1344, our pilot study identified a *yqiC* transposon mutant that cannot colonize and invade HEp-2 cells. Our subsequent validation study revealed that *yqiC*, which is located outside *Salmonella* pathogenicity islands (SPIs), is required for bacterial colonization and invasion of *S*. Typhimurium in various human cells. It is also required for flagellation, bacterial motility, and the postinfectious production of inflammatory cytokines in the human intestinal epithelium. These findings provide insight into the role of *yqiC* in the early pathogenesis of *Salmonella* in host cells and its role in increased virulence through the downregulation of *fimZ*–dominated type-1 fimbrial genes and the upregulation of SPI-1, SPI-2, and flagellar genes [4]. A study reported that similar to other *Salmonella* genes such as *stbC*, *invS*, *arcZ*, and *adhE*, *yqiC* can negatively regulate type 1 fimbriae expression [5].

YqiC, which is encoded by *yqiC*, is a small protein of *S*. Typhimurium that is localized at the cytoplasmic and membrane subcellular fraction [3]. This protein shares biophysical and biochemical properties with the Brucella membrane fusogenic protein (BMFP) superfamily and is a trimeric coiled-coil structure that induces membrane fusion activity in vitro [6]. The depletion of *yqiC* (*ubiK*) in *S*. *enterica* reduced its UQ levels to 18% of its wild-type strain, indicating that *yqiC* influences the biosynthesis efficiency of UQ, which is also called coenzyme Q and acts as an electron carrier [7]. UbiK binds to another UQ biogenesis factor UbiJ to form a heterotrimer UbiK–UbiJ complex that interacts with palmitoleic acid, which is a main lipid in *E*. *coli*; this protein works as an accessory factor that interacts with certain Ubi proteins to facilitate efficient aerobic (but not anaerobic) UQ-8 biosynthesis in *E* *coli* MG1655 and *S*. *enterica* 12023 [7]. In addition, because of the unique requirement of *ubiI* and *ubiK* for growing *E*. *coli* in oleate, they are a more suitable carbon source than succinate for inducing UQ biosynthesis that reduces the increased levels of reactive oxygen species (ROS) generated by long-chain fatty acid degradation [8].

The cellular respiration of bacteria has mainly been explored in *E* *coli*; however, research on its occurrence in *Salmonella* is limited. Cellular respiration converts chemical energy from nutrients for the synthesis of adenosine triphosphate (ATP) to fuel cellular activity, and it can be divided into glycolysis, tricarboxylatic acid (TCA) cycle, and electron transport pathways [9]. The respiratory chain (i.e. electron transport chain [ETC]) in a phospholipid membrane is catalyzed by similar membrane-bound protein complexes in most mitochondria and numerous types of bacteria. The composition of a cellular respiratory chain varies across species and exhibits less variation in the mitochondria of eukaryotic cells than in those of other cells. A chain usually comprises complexes I, III, and IV. Complex II (succinate-ubiquinone oxidoreductase) is not regarded as a member of the respiratory chain because it lacks a proton-motive function [10]. In contrast to the mitochondria in eukaryotic cells, most respiratory enzymes exit independently from each other in *E* *coli* without being organized into supercomplexes [11]. Compared with the four complexes in mitochondria, bacterial respiratory chains are considerably more diverse in terms of electron donors, carriers, and acceptors with identical general structures. Oxidative electron donating complexes (e.g., NADH:quinone oxidoreductases [NDH-1 cf. complex I and NDH-2], succinate dehydrogenases [cf. complex II], formate dehydrogenases, and hydrogenases) are mediated to reductive electron donor–acceptor complexes (including two types of terminal cytochrome oxidase [heme–copper oxidases, cf. complex IV, and cytochrome *bd*], nitrate reductases, nitrite reductases, fumarate reductases, tetrathionate reductases, and hydrogenases) through the electron carriers (quinones and cytochrome *c*) and intermediary complexes in specific species.

Quinone biosynthetic pathways in prokaryotes and eukaryotes are different. Through chorismate and various pathways, *E* *coli* and *Salmonella* synthesize three quinones with a side chain containing eight isoprene units (UQ-8, MK-8, and DMK-8) [11, 12]. MKs are the most frequently used electron carriers in bacterial respiratory chains; however, UQs can be used in specific alpha-, beta-, and gamma-proteobacteria [13]. The predominant quinone used for the aerobic growth of *E* *coli* is UQ; this is followed by DMK and MK [11]. In contrast to *E* *coli,* the biosynthesis and composition of quinones in *S.* *enterica* have received less attention from researchers. UQ-8 is the main quinone in the aerobic respiratory chain, and DMK-8 and MK-8 are the alternative electron carriers in the anaerobic respiration of *Salmonella* [14]. UQ biosynthesis under aerobic conditions requires oxygen, NADH, and flavoprotein, whereas MK biosynthesis requires 2-ketoglutarate, thiamine PPi, coenzyme A, and ATP as cofactors [12]. Our previous study demonstrated that *yqiC* is required for MK biosynthesis in *S*. Typhimurium [4]; by contrast, another study reported that *yqiC* is not involved in MK biosynthesis but influences the biosynthesis efficiency of UQ in *E* *coli* MG1655 and *S*. *enterica* 12,023 [7]. Whether the interaction between UQ and MK influences the virulence of bacterial colonization remains unclarified.

Few studies have examined the association of cellular respiration with bacterial colonization and invasion in early *Salmonella* infection and the corresponding mechanism. A study indicated that mutations in the *nuo* and *cyd* genes (which encode NDH-1 and cytochrome *d* oxidase, respectively) in ETCs suppress the anaerobic growth and colonization of *S*. Typhimurium in the alimentary tract of chickens [15]. In *S*. Typhimurium, mutations in *ubiA* and *ubiE* for UQ reduce flagella biogenesis, aerobic cellular respiration rates (oxygen consumption), and the membrane quinone pool (UQ and MK decreased, but DMK increased), which are partially increased by additional mutations in *nuoG*, *nuoM*, and *nuoN* for the NDH-1 components in a *ubiA*–*ubiE* double mutant, where MK and DMK are increased when NDH-1 transfers electrons from deamino-NADH to DMK or MK [14]. However, the interactions between NDH-1 and the UQ biosynthesis pathway are complex and may alter the level and composition of the quinone pool or the level and activity of NDH-1 enzymes. In addition, *ubiC* and *ubiA* are required for replicating *S*. Typhimurium within human cervical epithelial cells and murine colon enterocytes [16]. Our previous study demonstrated that the colonization-associated gene *yqiC* is required for MK biosynthesis in *S*. Typhimurium through its involvement in the ETC because the effects of *yqiC* are similar to those of NADH dehydrogenase, suggesting the involvement of MK and ETC in *yqiC* phenotyping [4]. By contrast, another study reported that the depletion of *ubiK* in *E* *coli* slightly increased MK-8 levels, suggesting that it has a minor effect on MK biosynthesis, although it is required for the proliferation of *S.* *enterica* in macrophages and virulence in mice [7]. Whether *yqiC* modulates the other *Salmonella* genes for regulating virulence factors and how *yqiC* participates in cellular respiration and oxidative stress warrant further clarification.

Therefore, we identified major *yqiC*-regulated genes and their involved pathways by comparing the whole genome transcriptomes of *S*. Typhimurium wild-type strain and its *yqiC*-deleted mutant, including the mRNA expression of the genes involved in SPIs, ETCs, and the biosynthesis of UQ and MK. Next, we performed RNA sequencing (RNA-seq) to clarify the effects of *yqiC* on *Salmonella* genes, used a Seahorse XFp Analyzer to study cell energy, and conducted a series of assays to explore the role of *yqiC* in energy metabolism, cellular respiration, ETCs, and ROS. To the best of our knowlegde, this is the first study to demonstrate the influence of *yqiC* on the unreported virulence in early salmonellosis and its contribution to cellular respiration and oxidative stress.

## Methods

### Bacterial strains and culture conditions

The wild-type *S*. Typhimurium strain SL1344 (SL1344), *yqiC*-deleted mutant strain (Δ*yqiC*), and *yqiC*-complemented Δ*yqiC* strain (Δ*yqiC*') were used in the present study. SL1344 was provided by Professor Duncan Maskell. The *S*. Typhimurium mutant strain (Δ*yqiC*) was created using the modified lambda(λ)-red recombinase method [17]. The Δ*yqiC*' was created using *yqiC*-specific primers to amplify the *yqiC*-coding sequence, which was then reversed and cloned into the pACYC184 vector to restore *yqiC* expression [4]. The *S*. Typhimurium strains SL1344, Δ*yqiC*, and Δ*yqiC*' were cultured in 2-mL antibiotic-free lysogeny broth (LB) medium and incubated in 5% CO_2_ and 95% air at 37 °C for 18 h as overnight cultures. The overnight cultures of SL1344, Δ*yqiC*, and Δ*yqiC*' were diluted at a 1:100 ratio into fresh LB broth; subsequently, they were incubated through shaking (225 rpm) in 5% CO_2_ and 95% medium at 37 °C for 2 h to obtain mid-log cultures for further analysis with the Seahorse XFp Analyzer. In addition, the overnight cultures of SL1344, Δ*yqiC*, and Δ*yqiC*' were grown aerobically and anaerobically at 37 °C, and mid-log cultures were obtained by shaking the incubated, 1:100-diluted overnight cultures in LB broth for 3–4 h to achieve an OD_600_ (i.e., optical density at 600 nm) of 0.7 for in vitro Caco-2 infection with RNA-seq and to perform DNA isolation with quantitative real-time (qRT) polymerase chain reaction (PCR) for the selected ETC genes.

### In vitro infection of *S*. Typhimurium and its* yqiC*-deleted mutant in Caco-2 cells

To identify significantly upregulated or downregulated genes in *S*. Typhimurium after the deletion of *yqiC*, an in vitro bacterial infection was conducted through the coculturing of SL1344 and Δ*yqiC* with Caco-2 cells, which were purchased from the Bioresource Collection and Research Center Taiwan (BCRC No. 67001, originally from ATCC No. HTB-37); the coculture was then seeded at a density of cells/T75 flask and maintained in complete Dulbecco’s modified Eagle’s medium (DMEM; 4500-mg/L glucose; Gibco) supplemented with 10% fecal bovine serum (Sigma), 0.1 mM nonessential amino acids (Sigma), 2 mM L-glutamine (Gibco), 1 mM sodium pyruvate (Gibco), and 0.01 mg/mL transferrin (Sigma) at 37 °C in 5% CO_2_. The medium was replaced with complete DMEM without fetal bovine serum 1 h before bacterial infection. Subsequently, 4-d-old confluent Caco-2 cells (1.04 × 10^7^ cells/T75 flask) were infected with the mid-logarithmic cultures of SL1344 and Δ*yqiC* (multiplicity of infection = 50) for 2 h. Thereafter, the DMEM containing the bacteria was centrifuged (13,000 × *g* for 2 min) to harvest cell pellets for subsequent RNA isolation. The experiments were performed independently in triplicate.

### RNA isolation and RNA sequencing

Bacterial RNA was isolated from the cell pellets of SL1344 and Δ*yqiC* after in vitro infection in Caco-2 cells, which was performed using the Total RNA Miniprep Purification Kit (GeneMark, Taichung, Taiwan) in accordance with the manufacturer’s protocol; the bacterial RNA was then processed for RNA-Seq by Welgene Biotech (Taipei, Taiwan) [18]. The isolated RNA samples were quantified using an ND-1000 spectrophotometer (Nanodrop Technology, Wilmington, DE, USA) at 260 nm, and their integrity was verified using a Bioanalyzer 2100 (Agilent Technologies, Santa Clara, CA, USA) with an RNA 6000 LabChip kit (Agilent Technologies). All the aforementioned procedures were performed in accordance with the Illumina protocol. Library preparation was then conducted using the TruSeq RNA Sample Prep Kit v2 (Illumina, San Diego, CA, USA) for 160-bp (single-end) sequencing. The RNA sequence was directly determined by employing sequencing-by-synthesis technology using the TruSeq SBS Kit, and raw sequences were acquired using Illumina GA Pipeline software CASAVA v1.8 (Illumina), which can generate 30 million reads per sample. Quantification for gene expression was computed as reads per kilobase of exon per million mapped reads (RPKM) [19]. The Cuffdiff tool (a part of the Cufflinks package) was used to calculate the expression fold changes and associated *q* values (false discovery rate-adjusted *p* values) for each gene between SL1344 and Δ*yqiC* [20]. The mean ratios for the expression of individual genes in Δ*yqiC* relative to SL1344 were expressed as log2 fold changes. In the present study, a log2 fold change ratio of > 1 or <  − 1 with a *q* value of < 0.05 was regarded as statistically significant.

### qRT-PCR for validation of RNA-seq analysis and for mRNA expression of genes encoding ETC enzymes

The total RNA samples isolated from the mid-log cultures of SL1344 and Δ*yqiC* were cleaned and purified using RNase-free DNase I (1 unit/1-µg RNA; NEB, Beverly, MA, USA). Subsequently, 0.5 µg of RNA was reverse transcribed to cDNA by using an iScript cDNA Synthesis Kit (BioRad, California, USA) in accordance with the manufacturer’s instructions. The oligonucleotide primer pairs specific to the target genes were designed using Primer3 and BLAST (http://www.ncbi.nlm.nih.gov/tools/primer-blast/); they included the 10 most significantly upregulated and 10 most significantly downregulated genes in the RNA-seq analysis (Additional file 1: Table S1), the five genes (i.e., *nuoE*, *ndh*, *sdhB*, *cyoC*, and *cydA*) encoding the ETC enzymes, and the housekeeping 16 s ribosomal RNA gene (Additional file 2: Table S2). Through the use of the Bio-Rad C100 Real-Time PCR System, 0.1 µg of cDNA was amplified in a 20-µL reaction solution containing 0.5 µM of each primer and 10 µL of iQTM SYBR Green Supermix (2 ×; BioRad) after we applied 40 cycles of enzyme activation at 95 °C for 3 min, denaturation at 95 °C for 30 s, annealing at 54 °C for 30 s, and extension at 72 °C for 30 s. The mRNA transcription levels were calculated using the 2^−ΔΔCt^ method as described in [21], and the expression levels of the housekeeping 16 s ribosomal RNA gene served as a basis for normalization. The mRNA expression levels of the selected genes in Δ*yqiC* were compared with those of the corresponding genes in SL1344 by using Student’s *t* test. In addition, the mRNA expression levels of the selected genes that represent the five ETC components were subjected to a pairwise comparison in the three aerobically and anaerobically grown *S* Typhimurium strains by performing Student’s *t*-test. The quantitative data were expressed as means ± standard errors of the mean (SEMs) of at least three measurements of fold change relative to the geometric mean of the normalized mRNA expression levels in *S*. Typhimurium SL1344. A *p* value of < 0.05 was regarded as statistically significant.

### Gene Ontology and Kyoto Encyclopedia of Genes and Genomes enrichment analyses for RNA-seq

The Cuffdiff output profiles in RNA-seq data were compared with the whole genome transcriptomes in *S*. Typhimurium SL1344 and Δ*yqiC* and further annotated through the addition of gene function descriptions and Gene Ontology (GO) terms; the reference genome and gene annotations were retrieved from the Ensemble database (https://www.ncbi.nlm.nih.gov/assembly/GCF_000210855.2). GO term enrichment and Kyoto Encyclopedia of Genes and Genomes (KEGG) pathways were applied to conduct the functional analysis of significantly regulated genes and identify *yqiC*-associated biological and functional themes through clusterProfiler V3.6 (https://bioconductor.riken.jp/packages/3.6/bioc/html/clusterProfiler.html), which includes cnetplot and emapplot (enrichMap). Cnetplot was used to determine the complex associations among the significantly regulated genes that exhibit potential biological complexity such that they each belong to multiple annotation categories. Emapplot was deployed to organize enriched terms into a network with connecting overlapping gene sets in which mutually overlapping gene sets cluster together. The KEGG (http://www.genome.jp/kegg/) was used to understand the interactions, reactions, and relation networks among cells, organisms, and ecosystems.

### ATP assay

To study the effect of *yqiC* on ATP production, an ATP assay of the overnight cultures of the *S*. Typhimurium SL1344, Δ*yqiC*, and Δ*yqiC*' strains was performed using a BacTiter-Glo Microbial Cell Viability Assay (Promega, Madison, WI, USA) in accordance with the manufacturer’s instructions. Finally, a GloMax Navigator Microplate Luminometer (Promega) was used to read the 96-well plates containing the samples, and their luminescence was recorded. The experiments were performed independently in triplicate. The generated ATP concentrations of Δ*yqiC* were statistically compared with those of SL1344 and Δ*yqiC*' by using Student’s *t* test. A *p* value of < 0.05 was regarded as statistically significant.

### Bacterial respiration assay, cell energy phenotype test, and glycolysis stress test

A bacterial respiration assay, glycolysis stress test, and cell energy phenotype test were performed by using a Seahorse XFp Extracellular Flux Analyzer (Seahorse Bioscience, North Billerica, MA, USA) and applying a modified version of the manufacturer’s instructions as described in another study [22]. On the day before the assays were conducted, a sensor cartridge of the XFp Analyzer was hydrated by filling each well of a miniplate with 200 μL of XFp Calibrant solution, filling the moats around the outside of each well with 400 μL of solution per chamber, and storing the cartridge assembly in a non-CO_2_ incubator at 37 °C overnight. Furthermore, overnight cultures of SL1344, Δ*yqiC*, and Δ*yqiC*' were prepared. On the day of the experiment, the miniplates used in the experiment were precoated with 50 mg/mL of poly-D-lysine for 30 min, and the wells were then rinsed twice with 200 μL of sterile distilled H_2_O and dried at room temperature for 20 min. Subsequently, the overnight cultures of SL1344, Δ*yqiC*, and Δ*yqiC*' were diluted at a 1:100 ratio in fresh LB broth and incubated with shaking at 37 °C for 2 h to achieve an OD_600_ of 0.2, and they were then dilated to 10 × the final OD_600_ of 0.02. Subsequently, 90 µL of the diluted cells were seeded at a concentration of 1.25 × 10^7^ cells/mL, and 90 μL of assay medium (5-mM TRIS [pH 7.6] with 2.5% glycerol, 150-mM NaCl, 5-μM ZnSO_4_, and 2% of 100% LB) was added to each miniplate well. In addition, 180 μL of prewarmed incubator assay medium was loaded into the wells without bacteria for use as background controls. The loaded microplates were centrifuged at 700 × *g* for 20 min to achieve cell attachment to the miniplate wells and allow for three assays to be conducted individually.

To assess the metabolic effects of *yqiC* and antibiotic stress on bacterial respiration, a bacterial respiration assay was performed independently in quadruplicate to quantitate the oxygen consumption rates (OCRs) and extracellular acidification rates (ECARs) as modified in other studies [23, 24]. Through the use of the Seahorse XFp Analyzer, OCR and ECAR measurements were obtained under incubation at 37 °C. To ensure uniform cellular seeding, basal OCRs and ECARs were measured for four cycles of 7 min before the injection of ampicillin (2.5 × 10^−3^ μg/mL, 5 × minimum inhibition concentration [MIC]) at 28 min, and they were quantitated every 7 min for the duration of the posttreatment experiment (200 min).

To detect metabolic switching from baseline to stressed status in live bacterial cells, the cell energy phenotype test was performed in eight independent experiments by using the Agilent Seahorse XFp Cell Energy Phenotype Test Kit. After baseline incubation for 30 min, 2 μM of oligomycin (an inhibitor of ATP synthase) and 1 μM of carbonyl cyanide-4 (trifluoromethoxy) phenylhydrazone (FCCP, an uncoupler of oxidative phosphorylation) were simultaneously injected with subsequent incubation for another 30 min, during which OCRs and ECARs were measured and used as baselines for stressed metabolic phenotypes.

To examine the effects of *yqiC* on glycolysis and oxidative phosphorylation, a glycolysis stress test was performed independently in triplicate by using the Agilent Seahorse XF Glycolysis Stress Test Kit Reagents of the Seahorse XFp Analyzer. First, cells were incubated in a medium without glucose or pyruvate, and ECAR measurements were obtained under incubation at 37 °C. After incubation for four cycles at 28 min, a saturation concentration of 10 mM glucose was injected to allow for the catabolism of glucose through the glycolytic pathway and ensue the rapid increase of ECAR, which was reported as the rate of glycolysis under basal conditions. After another four cycles of incubation at 54.4 min, 2 μM of oligomycin (an ATP synthase inhibitor) was injected to shift energy production to glycolysis and reveal the cellular maximum glycolytic capacity on the basis of the subsequent increase in ECAR. After another four cycles of incubation at 84.4 min, 50 mM of 2-deoxyglucose (2-DG; a glucose analog that inhibits glycolysis) was injected to induce a decrease in ECAR and determine the contribution of glycolysis in ECAR production; the difference between glycolytic capacity and glycolysis rate was defined as the glycolytic reserve. The extracellular acidification that occurs prior to glycose injection is the nonglycolytic acidification caused by nonglycolytic cell processes. ECARs were measured at every time point after each cycle over a duration of 173.2 min. In addition, glycolysis (maximum rate measurement before oligomycin injection − final rate measurement before glucose injection), glycolytic capacity (maximum rate measurement after oligomycin injection − final rate measurement before glucose injection), glycolytic reserve (glycolytic capacity − glycolysis), and glycolytic reserve as a percentage value (glycolytic capacity rate/glycolysis × 100) were also calculated and presented as parameters for output.

The obtained data pertaining to Δ*yqiC* and SL1344/Δ*yqiC*' were expressed as means ± SEMs and statistically compared using Student’s *t* test. A *p* value of < 0.05 was regarded as statistically significant.

### NADH/NAD^+^ assay and H_2_O_2_ assay

To investigate redox status, the concentrations of NADH and NAD^+^ were determined using an EnzyChrom NAD^+^/NADH Assay Kit (BioAssay Systems, Hayward, CA, USA) in accordance with the manufacturer’s instructions as described in other studies [25, 26]. To evaluate intracellular oxidative stress, H_2_O_2_ production was measured by using an Amplex Red Hydrogen Peroxide/Peroxidase Assay Kit (Invitrogen, Carlsbad, CA, USA) and by applying a modified version of the manufacturer’s instruction as described in another study [22]. In brief, the mid-log cultures of SL1344, Δ*yqiC*, and Δ*yqiC*' with an OD_600_ of 0.7 (approximately 1 × 10^8^ colony-forming unit/mL) were centrifuged at 14,000 × *g* into pellets and resuspended in assay medium for the subsequent processing of the two aforementioned assays in accordance with their individual protocols. The completed microplate wells containing the standards, controls, and bacterial samples were incubated with light protection at room temperature for 30 min, after which a final fluorescence reading was taken. Through the use of a SpectraMax reader (Molecular Devices, Sunnyvale, CA), the concentrations of NADH and NAD^+^ and those of H_2_O_2_ were determined at 565 and 590 nm, respectively; calculations were performed in accordance with the standard curves that were plotted using the serially diluted standard solutions, as presented using Microsoft Office Excel 2013. NADH/NAD^+^ ratios were also calculated. The quantitated data from the independent experiments performed in triplicate were expressed as means ± SEMs, and the results for Δ*yqiC* and SL1344/Δ*yqiC*' were statistically compared using Student’s *t* test. A *p* value of < 0.05 was regarded as statistically significant.

## Results

### *yqiC* inactivates the upregulation of genes in aspartate carbamoyl transferase, type 1 fimbriae, iron–sulfur assembly, and NADH dehydrogenase during the early colonization of *S*. Typhimurium in Caco-2 cells

**Fig. 1:**
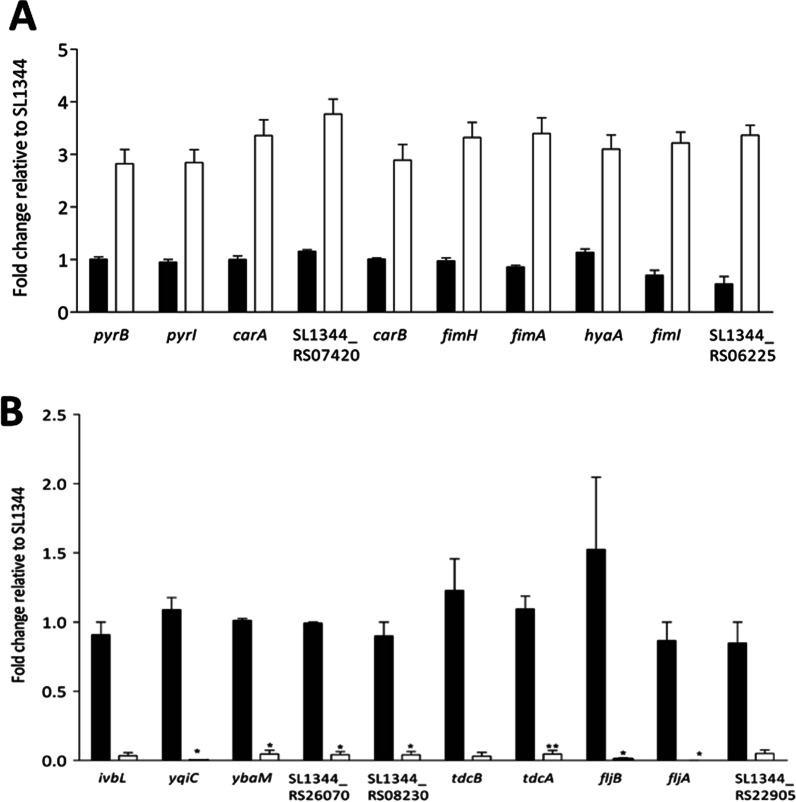
Quantitative real-time polymerase chain reaction for validating the 10 most significantly upregulated genes (**A**) and the 10 most significantly downregulated genes (**B**) of Δ*yqiC* relative to *S*. Typhimurium SL1344 after in vitro infection with Caco-2 cells for 2 h in triplicate

An RNA-seq analysis identified 117 significantly upregulated genes of Δ*yqiC* relative to SL1344 after in vitro infection with Caco-2 cells for 2 h (Additional file 3: Table S3A). The 10 most significantly upregulated genes (Table 1A) were selected for qRT-PCR to validate their upregulation after *yqiC* depletion (Fig. 1A). *yqiC* depletion resulted in the upregulation of the genes in several functional groups, including those involved in aspartate carbamoyltransferase (*pyrB*, *pyrI*, *pyrE*, and *pyrD*, and *pyrC*), type 1 fimbriae (*fimH*, *fimA*, *fimI*, *fimD*, *fimC*, *fimF*, *fimZ*, and *fimW*), RNA polymerase sigma factor (*rpoS*), SL1344_RS12565 (*rpoE*-regulated lipoprotein), cytochromes (SL1344_RS06220 and *aapB*), spermidine/putrescine transporter substrate-binding protein (*potF*), iron–sulfur assembly (*yfhP*, *yfhF*, *fdx*, *nifU*, and *yhgI*), osmoprotectant ABC transporter substrate-binding proteins (SL1344_RS07420, SL1344_RS07415, and SL1344_RS07410), NADH dehydrogenase (*ndh*), and some hypothetical proteins.

**Table 1:** RNA-seq analysis revealed the 20 most significantly upregulated genes (**A**) and the 20 most significantly downregulated genes (**B**) of Δ*yqiC* relative to *S*. Typhimurium SL1344 after in vitro infection with Caco-2 cells for 2 h

No.	SL1344 gene name (or locus tag)	LT2 gene name	Description	Log2 fold change (Δ*yqiC*/SL1344)	*q* value
**A**					
1	*pyrB*	*pyrB*	Aspartate carbamoyltransferase catalytic subunit	4.205	0.001601
2	*pyrI*	*pyrI*	Aspartate carbamoyltransferase regulatory subunit	3.928	0.001601
3	*carA*	*carA*	Carbamoyl phosphate synthase small subunit	3.267	0.001601
4	SL1344_RS07420	STM1493	Osmoprotectant ABC transporter substrate-binding protein OsmX	2.661	0.012098
5	*carB*	*carB*	Carbamoyl phosphate synthase large chain	2.358	0.001601
6	*fimH*	*fimH*	Fimbrial adhesin FimH	2.279	0.001601
7	*fimA*	*fimA*	Type 1 fimbrial protein subunit FimA	2.059	0.001601
8	*hyaA*	STM1786	[Ni/Fe] hydrogenase small subunit	2.015	0.006092
9	*fimI*	*fimI*	Type 1 fimbrial protein subunit FimI	1.998	0.001601
10	SL1344_RS06225	STM1254	Hypothetical protein	1.974	0.001601
11	SL1344_RS25015	–	Hypothetical protein	1.931	0.001601
12	*pyrE*	*pyrE*	Orotate phosphoribosyltransferase	1.923	0.001601
13	*fimD*	*fimD*	Outer membrane usher protein	1.899	0.001601
14	*iscS*	*nifS*	IscS subfamily cysteine desulfurase	1.899	0.001601
15	*glnK*	*glnK*	Nitrogen regulatory protein P-II 2	1.882	0.007075
16	*pdhR*	*pdhR*	Pyruvate dehydrogenase complex repressor	1.881	0.001601
17	*fimC*	*fimC*	Fimbrial chaperone protein FimC	1.862	0.001601
18	SL1344_RS07595	STM1527	Hypothetical protein	1.814	0.001601
19	*ygaC*	*ygaC*	Hypothetical protein	1.805	0.001601
20	*fimF*	*fimF*	Fimbrial-like protein FimF	1.799	0.001601
**B**					
1	*ivbL*	*ivbL*	*ilvB* operon leader peptide IvbL	− 25.492	0.022391
2	*yqiC*	*yqiC*	Hypothetical protein	− 23.906	0.001601
3	*ybaM*	*ybaM*	DUF2496 domain-containing protein	− 21.810	0.002902
4	SL1344_RS26070	–	Hypothetical protein	− 19.967	0.022391
5	SL1344_RS08230	–	Hypothetical protein	− 19.143	0.022391
6	*tdcB*	*tdcB*	Serine/threonine dehydratase	− 4.197	0.001601
7	*tdcA*	*tdcA*	Transcriptional regulator TdcA	− 3.979	0.038047
8	*fljB*	*fljB*	Flagellin FliC	− 3.689	0.001601
9	*fljA*	*fljA*	Phase 1 flagellin transcriptional repressor	− 3.536	0.002902
10	SL1344_RS22905	STM4465	Ornithine carbamoyltransferase	− 3.419	0.011296
11	SL1344_RS12015	STM2342	PTS ascorbate transporter subunit IIC	− 3.350	0.001601
12	*tdcD*	*tdcD*	Propionate kinase	− 3.106	0.001601
13	*cadA*	*cadA*	Lysine decarboxylase CadA	− 2.988	0.001601
14	SL1344_RS22110	STM4306	Dimethylsulfoxide reductase, chain B	− 2.979	0.001601
15	SL1344_RS22105	STM4305	Dimethyl sulfoxide reductase subunit A	− 2.883	0.001601
16	*tdcC*	*tdcC*	Threonine/serine transporter TdcC	− 2.869	0.001601
17	*cadB*	*cadB*	Arginine:agmatine antiporter	− 2.809	0.001601
18	SL1344_RS23095	STM4502	DUF1062 domain-containing protein	− 2.641	0.007995
19	*pndA*	STM0886	Protein PndA	− 2.557	0.001601
20	SL1344_RS25925	–	Hypothetical protein	− 2.509	0.001601

### *yqiC* is required for expressing *ilvB* operon, flagellin, *tdc*ABCD, *dms*AB, the cytochrome c family, and formate dehydrogenase during the early colonization of *S*. Typhimurium in Caco-2 cells

An RNA-seq analysis identified 291 significantly downregulated genes of Δ*yqiC* relative to SL1344 after in vitro infection with Caco-2 cells for 2 h (Additional file 3: Table S3B). The 10 most significantly downregulated genes (Table 1B) were selected for qRT-PCR to validate their downregulation after *yqiC* depletion (Fig. 1B). The most significantly downregulated gene was *ilvL* (− 25.5 log2 fold change). In addition, *yqiC* depletion leads to the downregulation of the genes involved in flagellin (*fljB* and *fljA*), threonine ammonia-lyase *tdc* family (*tdcB*, *tdcA*, *tdcD*, *tdcC*, and *tdcE*), anaerobic dimethylsulfoxide reductase (*dmsA*, *dmsB*, and *dmsC*) and various anaerobic enzymes (*glpA*, SL1344_RS00185, *dcuC*, *nrdD*, and *nrdG*), the cytochrome c family (*nrfA*, SL1344_RS19665, *ccdA*, and *napC*), formate dehydrogenase (*fdhF*), and several hypothetical proteins.

### *yqiC* is required for expressing mainly SPI-1 genes and specific SPI-4, SPI-5, and SPI-6 genes but diversely regulates SPI-2 and SPI-3 gene expression

*yqiC* is required for the expression mainly SPI-1 genes and specific SPI-4, SPI-5, and SPI-6 genes. Nine SPI-1 genes (i.e., *sopA*, *sopD*, *sopE*, *prgH*, *sptP*, *sipD*, *sipC*, *sicA*, and *sipA*) were identified from the 291 significantly downregulated genes (Additional file 5: Table S5). By contrast, the depletion of *yqiC* diversely regulated the genes located within SPI-2 and encoding SPI-2 T3SS effectors, including the significant downregulation of *spvB*, *ttrA*, and *ttrS*, but also the significant upregulation of *sseE* and *sscA*. The depletion of *yqiC* significantly downregulated *ydiA* expression but caused a nearly significant upregulation for *mgtC* in SPI-3. The depletion of *yqiC* in *S*. Typhimurium resulted in a significant downregulation of *siiD* in SPI-4, *pipC* in SPI-5, and *safA* and *sciC* in SPI-6 (Additional file 5: Table S5).

### *yqiC* is involved in the expression of genes for *Salmonella* infection, bacterial invasion of epithelial cells, pathogenesis in extracellular region, cell adhesion, pili, and fimbriae

**Fig. 2:**
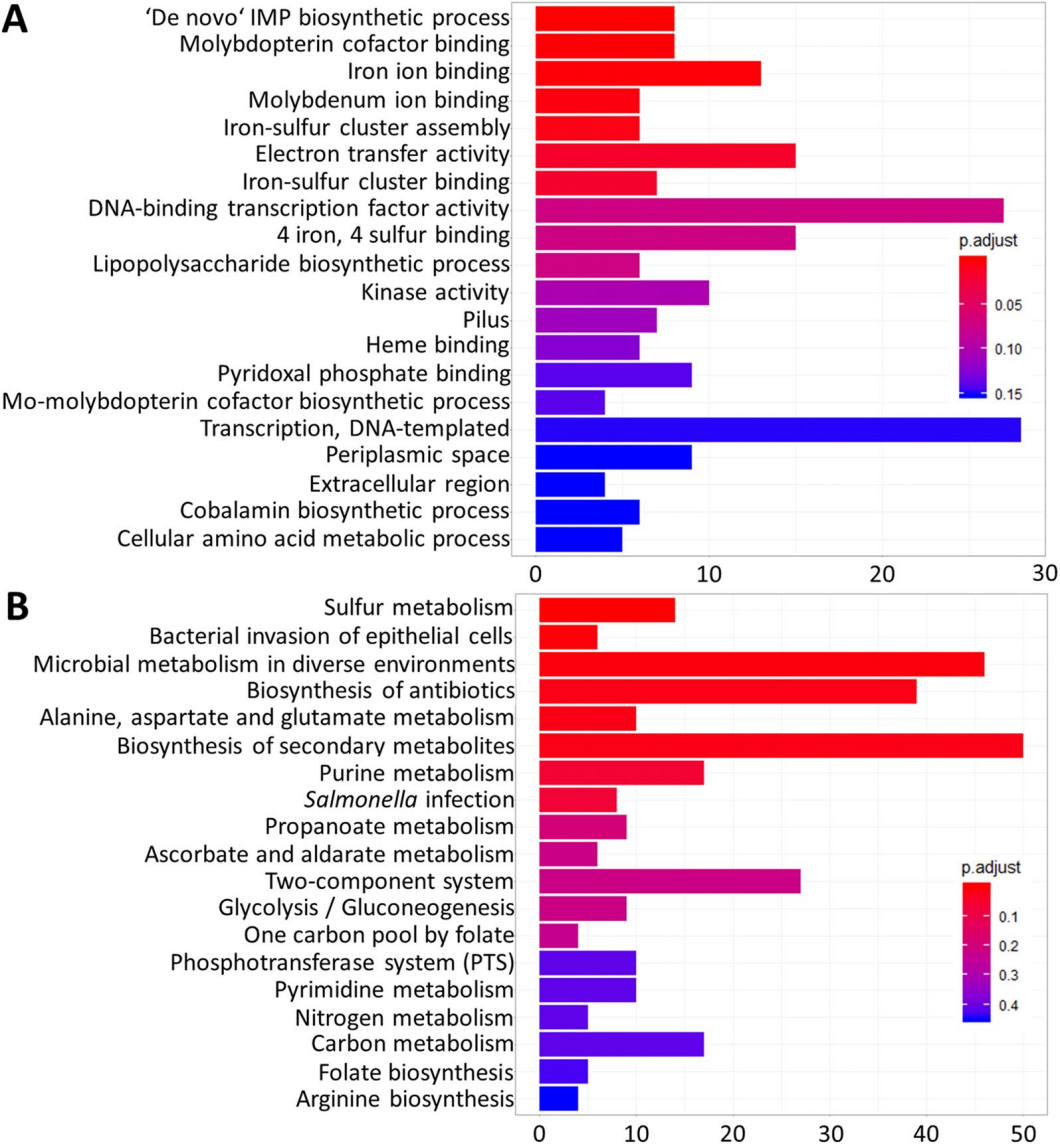
Top 20 GO terms (**A**) and 19 KEGG pathways (**B**) of *S*. Typhimurium that were most significantly enriched after *yqiC* depletion. The *x*-axis indicates gene numbers, and the *y*-axis indicates GO terms or KEGG pathways

**Fig. 3:**
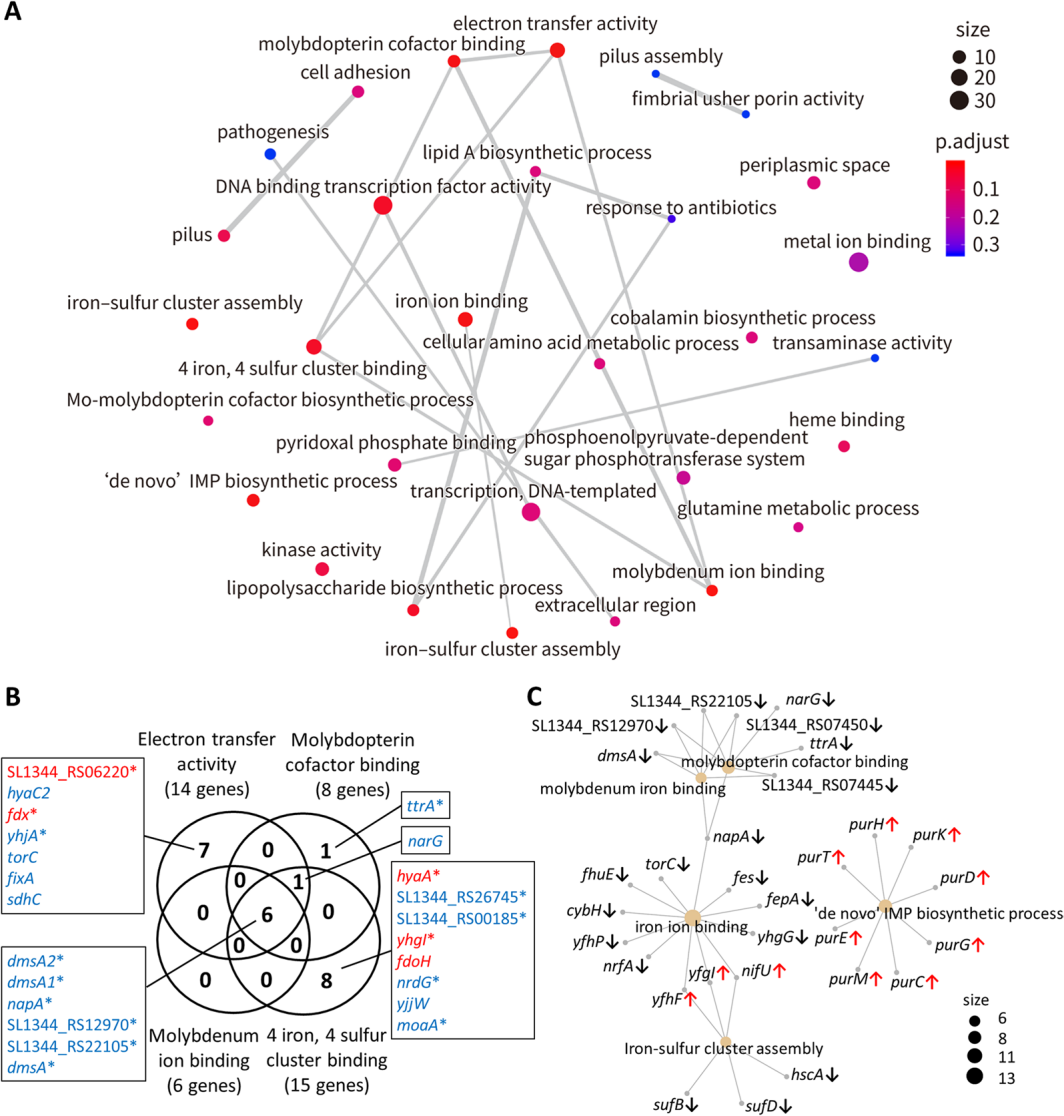
Emapplot (**A**), main emapplot gene clusters associated with electron transfer activity (**B**), and cnetplot (**C**) of GO enrichment analyses of RNA-seq of Δ*yqiC* relative to *S*. Typhimurium SL1344. Sizes of circles represent numbers of genes involved in 30 functional groups. Color shifts from blue to red indicate shifts from low significance to high significance (**A**, **C**). Upregulated and downregulated genes in a Venn diagram are highlighted in red and blue, respectively; an asterisk indicates statistical significance as defined in this study (**B**)

An RNA-seq analysis identified nine significantly downregulated SPI-1 genes after the depletion of *yqiC* in *S*. Typhimurium and interactions with Caco-2 cells (Additional file 5: Table S5). A GO enrichment analysis revealed that *yqiC* significantly regulated the seven genes responsible for pili and the four genes for extracellular region pathogenesis (Fig. 2A) that are associated with early interactions between *Salmonella* and host cells. In the GO term of the pilus, *yqiC* suppresses the expression of *fimA*, *fimI*, *fimH*, and *fimF*, which encode type 1 fimbrial proteins; however, it is required for expressing *stcA*, *stdA*, and *sthD*, which encode fimbrial proteins (Additional file 4: Table S4). In the GO term of the extracellular region, *yqiC* is required for expressing *sopE*, *sipA*, and *sipC*, which encode four SPI-1 T3SS effectors or complex proteins, and for *fljB* encoding flagellin (Additional file 7: Table S7); these genes are all key *Salmonella* virulence factors and were also identified in the list of significantly downregulated genes (Additional file 3: Table S3). An emapplot analysis (Fig. 3A) revealed the linkage between extracellular region (*sopE*, *fljB*, *sipA*, and *sipC*) and pathogenesis (*sigE*, *sopE*, *sipA*, *sipD*, *sipC*, and *sopD*) and the association between pilus and cell adhesion (both comprising *stcA*, *stdA*, *sthD*, *fimA*, *fimI*, *fimH*, and *fimF*). Furthermore, the two mutually linked clusters, pilus assembly and fimbrial usher porin activity (Fig. 3A), consisted of downregulated *stcC* and *stdB* and upregulated *fimD* (Additional file 7: Table S7), indicating their involvement in the early expression of fimbrial proteins and type 1 fimbriae. Similarly, a KEGG analysis revealed that *yqiC* is required for expressing virulence genes during *Salmonella* infection and the bacterial invasion of epithelial cells, including *sipC* and *sipB* (encoding translocons), *fliC*, *fljB*, *sipA*, *sipC*, *sipD*, *sopD*, *sopE*, and *sptP* (encoding SPI-1 effectors), *spvB* (encoding SPI-2 effectors), and *nrfA* (encoding NrfA for NO detoxification) (Additional file 12: Fig. S2, Additional file 13: Fig. S3).

### *yqiC* contributes to *menD* expression in MK biosynthesis

Among the 15 known *ubi* genes that are involved in UQ biosynthesis, *ubiA* and *ubiD* (*yigC*) exhibited an upregulation trend after the depletion of *yqiC* in *S*. Typhimurium (Additional file 6: Table S6A). Among the nine known *men* genes involved in MK biosynthesis, *menD* was significantly downregulated after the depletion of *yqiC* in *S*. Typhimurium (Additional file 6: Table S6B), demonstrating that *yqiC* contributed to the expression of *menD* in MK biosynthesis.

### *yqiC* is involved in electron ion transfer through the binding of molybdenum iron, iron ions, and iron–sulfur cluster

The most significantly enriched GO terms include the de novo inosine monophosphate (IMP) biosynthetic process, molybdopterin cofactor binding, iron ion binding, molybdenum ion binding, electron transfer activity, and iron–sulfur cluster assembly/binding (Fig. 2A). In addition, a KEGG enrichment analysis revealed that *yqiC* is required for gene expression in sulfur metabolism as the most significantly involved pathway (Fig. 2B), particularly *cysJ* in assimilatory sulfate reduction, *ttrA* in tetrathionate reduction, and *dmsABC* in anaerobic dimethyl sulfoxide reduction (Additional file 11: Fig. S1). These findings indicate that *yqiC* significantly influences electron ion transfer and the metabolism of molybdenum, iron, and sulfur.

The emapplot of a GO enrichment analysis of *yqiC* depletion in *S*. Typhimurium SL1344 revealed the effects of *yqiC* on 30 gene clusters (Additional file 7: Table S7), including the *yqiC*–associated gene cluster in electron transfer activity that comprises two significantly upregulated genes (SL1344_RS06220 and *fdx*) and seven significantly downregulated genes (*dmsA2*, *dmsA1*, *napA*, SL1344_RS12970, *yhjA*, SL1344_RS22105, and *dmsA*). The main network orchestrated electron transfer activity, molybdopterin cofactor binding, molybdenum ion binding, and 4 iron, 4 sulfur cluster binding (Fig. 3A, B). Given the complex interactions among the genes involved in this network, the six genes (*dmsA2*, *dmsA1*, *napA*, SL1344_RS12970, SL1344_RS22105, and *dmsA*) that were simultaneously and significantly downregulated comprise the core genes in the aforementioned four GO terms after the depletion of *yqiC* (Fig. 3B). By contrast, only four genes were significantly upregulated, namely SL1344_RS06220 (encoding cytochrome b) and *fdx* (encoding 2Fe-2S type ferrodoxin), which are involved in electron transfer activity, and *hyaA* (encoding hydrogenase-1 small subunit) and *yhgI* (encoding hypothetical protein), which are involved in 4 iron, 4 sulfur cluster binding (Fig. 3B). In addition, *yqiC* was associated with the linkage between iron ion binding and iron–sulfur cluster assembly (Fig. 3A). Collectively, *yqiC* significantly regulated the ion binding process and electron transfer activity through reciprocal interactions in the expression of the aforementioned genes.

The cnetplot of the GO enrichment analysis of the RNA-seq of Δ*yqiC* relative to *S*. Typhimurium SL1344 revealed five major clusters of significantly regulated genes and their connections to each other (Fig. 3C). The depletion of *yqiC* significantly upregulated the eight *pur* genes involved in the de novo IMP biosynthetic process (Additional file 4: Table S4) and functioned independently without connection to the other four clusters (Fig. 3C). *yqiC* depletion downregulated the genes of the two clusters involved in molybdenum ion binding and molybdopterin cofactor binding, both of which were linked with *dmsA* and *napA* (encoding nitrate reductase subunits) and SL1344_RS07445, SL1344_RS07450, SL1344_RS12970, and SL1344_RS22105 (encoding dimethyl sulfoxide reductase and its subunits). Through *napA*, the two aforementioned clusters connected with iron ion binding and formed further links with iron–sulfur cluster assembly through *yfhF*, *nifU*, and *yhgI*, which were negatively regulated by *yqiC* (Additional file 5: Table S5, Additional file 7: Table S7).

### *yqiC* is extensively associated with carbohydrate and energy metabolism

A KEGG enrichment analysis of the RNA-seq of *S*. Typhimurium after *yqiC* depletion identified 19 KEGG pathways that involve *yqiC* (Fig. 2B and Additional file 8: Table S8). A further investigation of the KEGG modules of *S*. Typhimurium SL1344 (https://www.genome.jp/kegg-bin/show_organism?menu_type=pathway_modules&org=sey) revealed that *yqiC* is extensively associated with two main modules: carbohydrate metabolism and energy metabolism. The KEGG pathways of glycolysis/gluconeogenesis, carbon metabolism, and microbial metabolism in diverse environments; ascorbate and aldarate metabolism; and the biosynthesis of secondary metabolites (Additional file 14: Fig. S4, Additional file 15: Fig. S5, Additional file 16: Fig. S6, Additional file 17: Fig. S7, Additional file 18: Fig. S8) were involved in the module of carbohydrate metabolism. By contrast, sulfur metabolism, microbial metabolism in diverse environments, and nitrogen metabolism (Additional file 11: Fig. S1, Additional file 16: Fig. S6, Additional file 19: Fig. S9) were involved in the module of energy metabolism.

### *yqiC* contributes to efficient ATP generation in *S*. Typhimurium

**Fig. 4:**
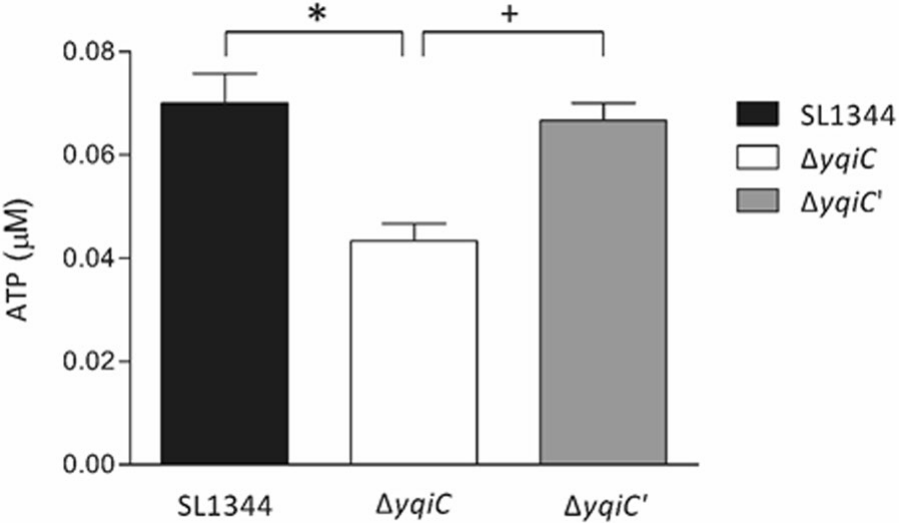
ATP assays performed independently in triplicate. ATP generation significantly decreased in *S*. Typhimurium Δ*yqiC* relative to paternal wild-type SL1344 (*p < 0.05) and *yqiC*-complemented strain Δ*yqiC*' (^+^*p* < 0.05)

An ATP assay was performed to further clarify the role of *yqiC* in energy metabolism in *S*. Typhimurium, and it revealed that the ATP concentrations in *S*. Typhimurium Δ*yqiC* were significantly lower than those in wild-type SL1344, and they were restored to a level similar to that of SL1344 after the complementation of *yqiC* in Δ*yqiC*' (Fig. 4). Therefore, *yqiC* significantly contributes to efficient ATP production in *S*. Typhimurium.

### *yqiC* is required for oxygen consumption and extracellular acidification in *S*. Typhimurium regardless of antibiotic stress

**Fig. 5:**
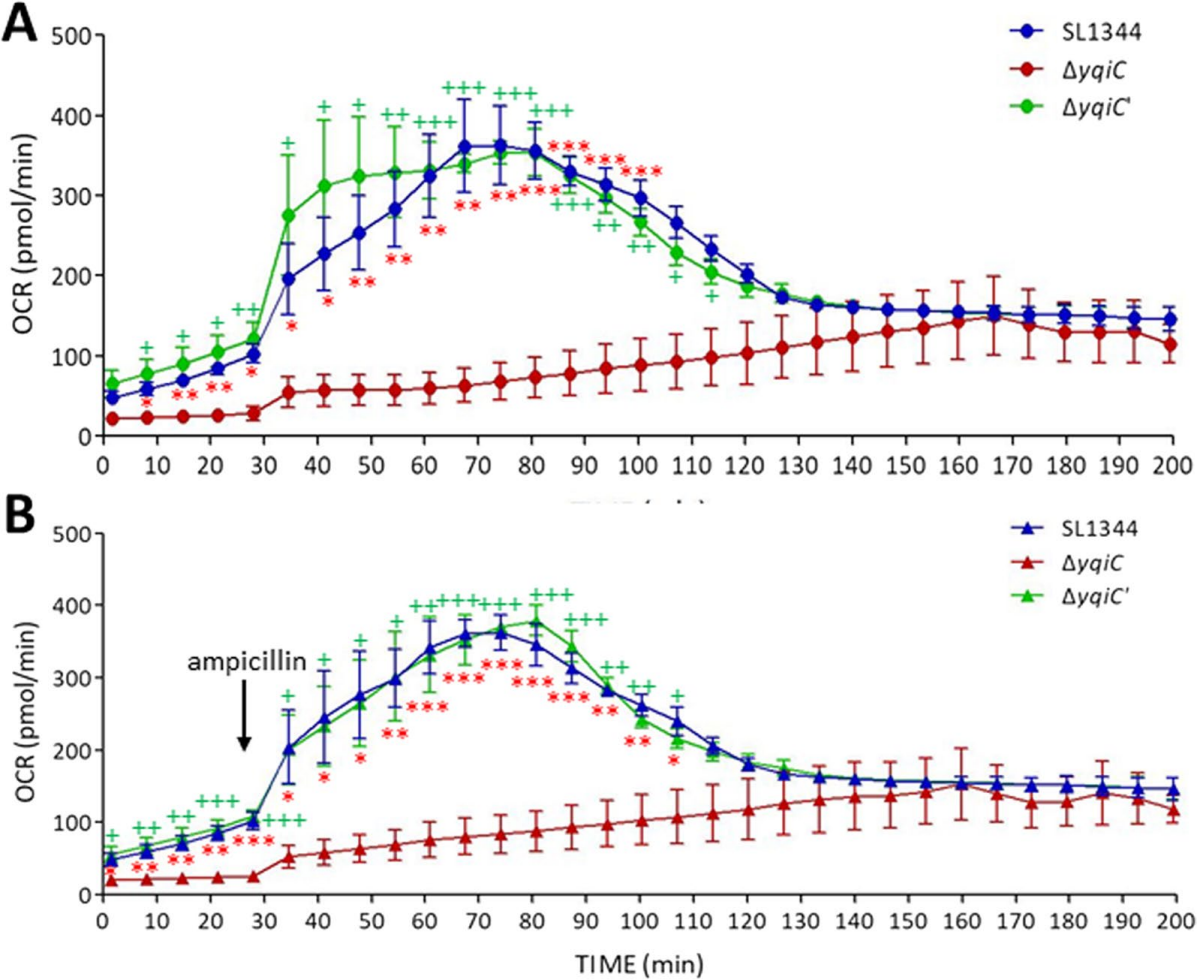
OCR assays performed in quadruplicate reveal significantly lower OCRs of *S*. Typhimurium Δ*yqiC* during the first 15 cycles after incubation relative to those of its paternal wild-type strain SL1344 and *yqiC*-complemented strain Δ*yqiC*'. OCRs obtained in the absence of ampicillin (**A**) and following injection of ampicillin at 28 min (**B**). *p < 0.05, **p < 0.01, and ***p < 0.001 for comparisons between Δ*yqiC* and SL1344; ^+^*p* < 0.05, ^++^*p* < 0.01, and ^+++^*p* < 0.001 for comparisons between Δ*yqiC* and Δ*yqiC*'

**Fig. 6:**
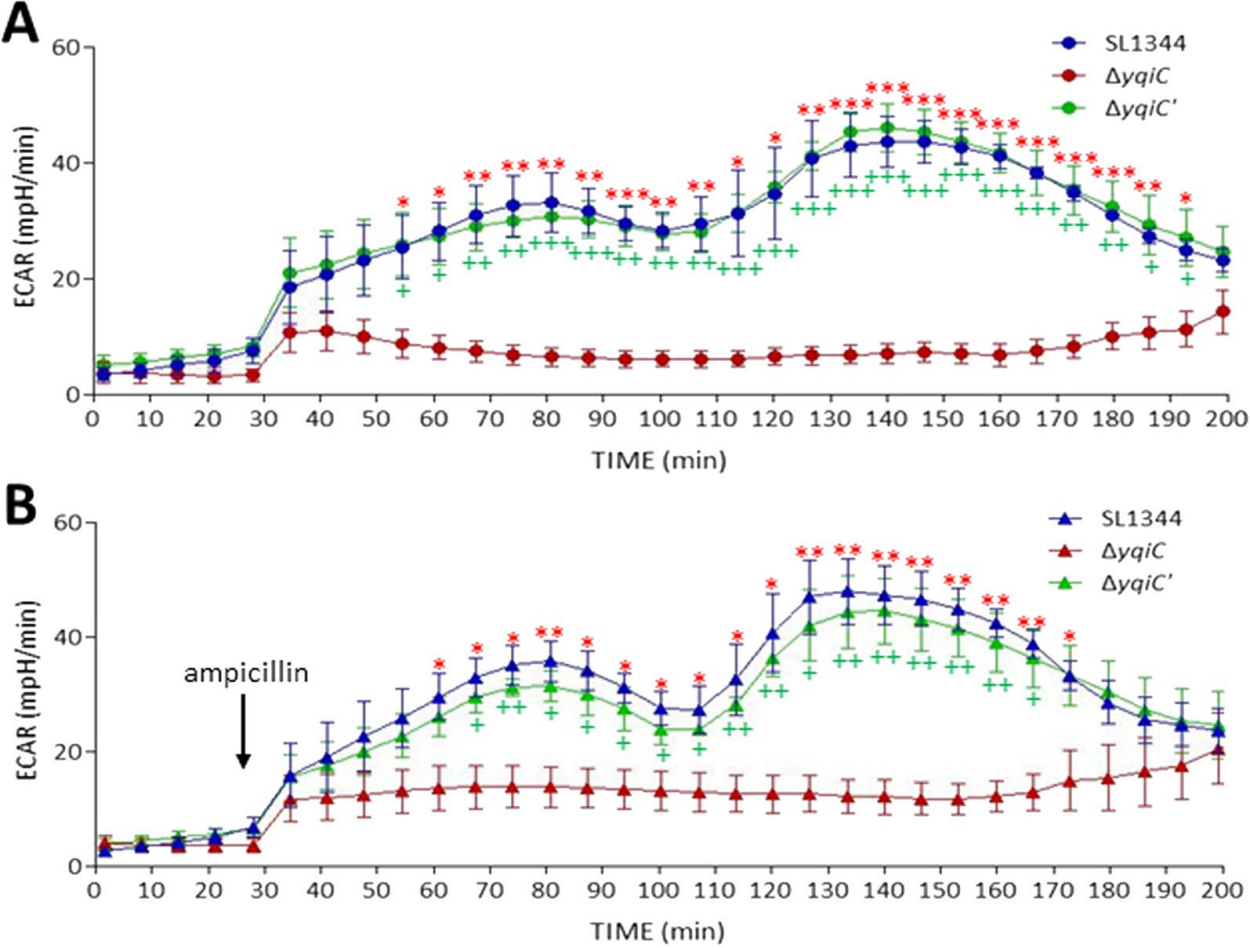
ECAR assays performed independently in quadruplicate reveal significantly lower ECARs of *S*. Typhimurium Δ*yqiC* from the 9th to 26th cycle after incubation relative to those of its paternal wild-type strain SL1344 and *yqiC*-complemented strain Δ*yqiC*'. ECARs obtained in the absence of ampicillin (**A**) and following injection of ampicillin at 28 min (**B**) (*p < 0.05 and **p < 0.01 for comparisons between Δ*yqiC* and SL1344; ^+^*p* < 0.05 and ^++^*p* < 0.01 for comparisons between Δ*yqiC* and Δ*yqiC*')

A bacterial respiration assay was performed to measure OCRs (i.e., the rate of cellular respiration), and it revealed significantly lower OCRs for Δ*yqiC* at 7 min and 105 min after incubation relative to those for SL1344 and Δ*yqiC*' (Fig. 5A). Ampicillin was injected to induce antibiotic stress after the fourth cycle in the Seahorse XFp Analyzer, and the significant difference between the OCR of Δ*yqiC* and those of SL1344 and Δ*yqiC*' remained unchanged from the first to fifteenth cycle (Fig. 5B), indicating that antibiotic stress minimally influenced the effect of *yqiC* on oxygen consumption in *S*. Typhimurium. Another energy pathway was examined by measuring ECAR (i.e., the rate of cell glycolysis), and *yqiC* was revealed to have significantly reduced the ECAR of Δ*yqiC* from the 9th to 26th cycle in the Seahorse XFp Analyzer; notably, the effect of *yqiC* on ECARs was prolonged and exhibited a later onset relative to its effect on OCRs (Fig. 6A). The injection of ampicillin did not significantly influence the differences between the ECAR of Δ*yqiC* and those of SL1344 and Δ*yqiC*' (Fig. 6B). Although SL1344 and Δ*yqiC*' both exhibited OCRs as monophasic waveforms and ECARs as biphasic waveforms, the depletion of *yqiC* considerably suppressed both energy phenotypes irrespective of ampicillin stress.

### *yqiC* is required for maintenance in cellular respiration and metabolic potential under energy stress

**Fig. 7:**
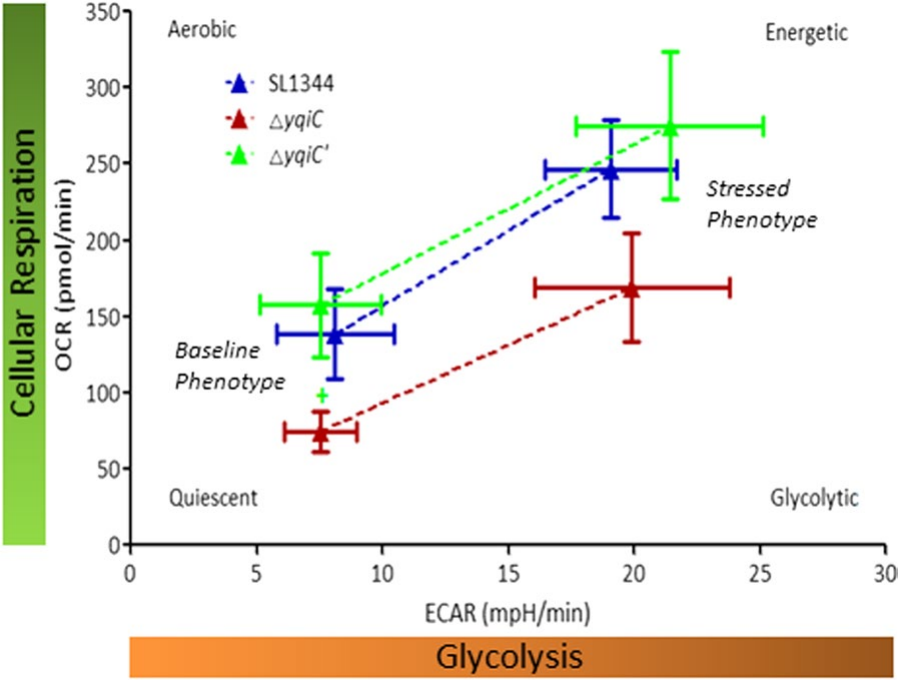
Cell phenotype energy test of *S*. Typhimurium SL1344, Δ*yqiC*, and Δ*yqiC'* as performed by using a Seahorse XFp Analyzer for eight independent experiments; the results indicate metabolic switching after *yqiC* depletion in *S*. Typhimurium (^+^*p* < 0.05 for comparisons between Δ*yqiC* and Δ*yqiC*')

A cell energy phenotype test was conducted to examine the effect of *yqiC* on the two major energy producing pathways of a cell affected by energy hunger, and it revealed a metabolic switching trend involving decreasing oxygen consumption after the depletion of *yqiC* in *S*. Typhimurium; significant differences between *S*. Typhimurium Δ*yqiC* and Δ*yqiC*' were detected in both basic and stressed phenotypes. The OCRs of *S*. Typhimurium Δ*yqiC* were lower than those of *S*. Typhimurium SL1344 and Δ*yqiC*' before and after the implementation of an in vitro energy stress intervention, which was achieved through the simultaneous use of oligomycin and FCCP (Fig. 7). However, the ECARs of Δ*yqiC* were not significantly different from those of SL1344 and Δ*yqiC*', indicating that *yqiC* mainly depended on cellular respiration under energy stress to maintain its metabolic potential, which reflects its cellular ability to meet energy demands.

### *yqiC* is crucial for glycolysis, glycolytic capacity, and glycolytic reserve

**Fig. 8:**
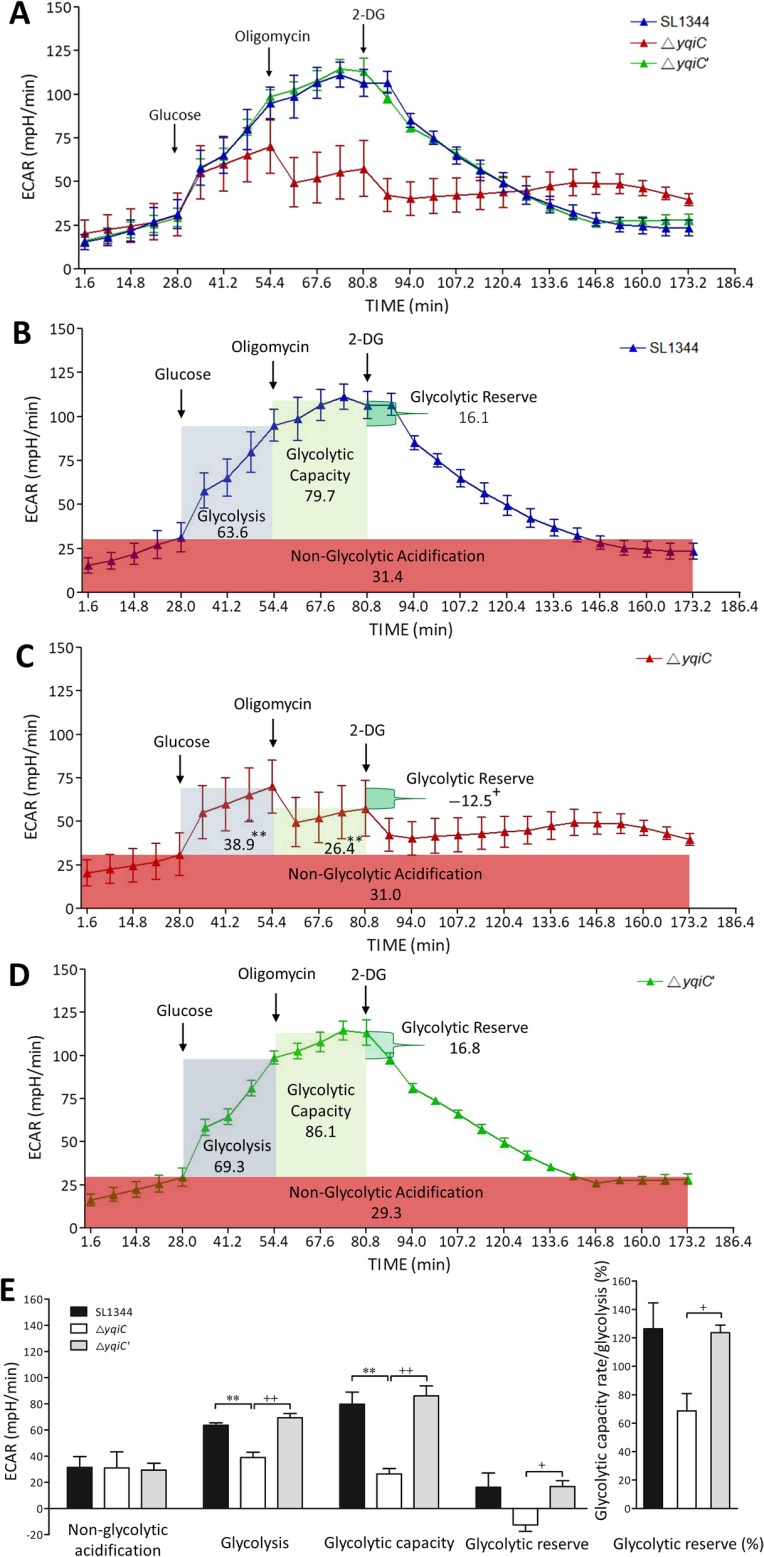
Glycolysis stress tests of *S*. Typhimurium SL1344, Δ*yqiC*, and Δ*yqiC*' as performed independently in triplicate using the Seahorse XFp Analyzer; the figure shows ECARs at various time points and profile parameters after *yqiC* depletion in *S*. Typhimurium. **p < 0.01 for comparisons between Δ*yqiC* and SL1344; ^+^*p* < 0.05 and ^++^*p* < 0.01 for comparisons between Δ*yqiC* and Δ*yqiC*'

A glycolysis stress test revealed that the ECARs of *S*. Typhimurium decreased after the depletion of *yqiC* and injection of glucose in the Seahorase XFp Analyzer after the 5th cycle; the test also revealed that the ECARs of Δ*yqiC* remained low following the injection of oligomycin and 2-DG until the 17th cycle (Fig. 8A). Other calculated profile parameters indicated significantly decreased glycolysis and glycolytic capacityin Δ*yqiC* relative to SL1344 andΔ*yqiC*'; and significantly decreased glycolytic reserve in Δ*yqiC* relative to Δ*yqiC*' (Fig. 8B–E). Collectively, the aforementioned findings indicate that *yqiC* is crucial for glycolysis, glycolytic capacity, and glycolytic reserve.

### *yqiC* is required for expressing *ndh*, *cydA*, *nuoE*, and *sdhB* but suppresses the upregulation of *cyoC* in the ETC of *S*. Typhimurium (particularly under anaerobic conditions)

**Fig. 9:**
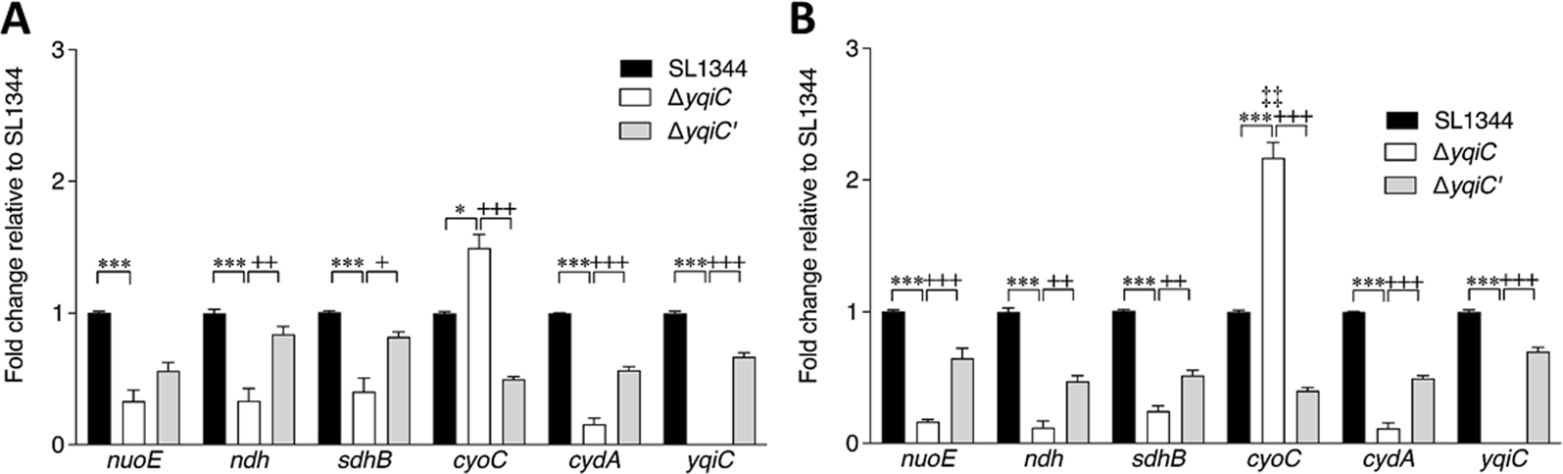
qRT-PCR for mRNA expression of five representative genes involved in ETC and *yqiC* when *S*. Typhimurium SL1344, Δ*yqiC*, and Δ*yqiC'* are cultured independently in quadruplicate under aerobic (**A**) and anaerobic (**B**) conditions (*p < 0.05, **p < 0.01, and ***p < 0.001 for comparisons between Δ*yqiC* and SL1344; ^+^*p* < 0.05, ^++^*p* < 0.01, and ^+++^*p* < 0.001 for comparisons between Δ*yqiC* and Δ*yqiC*'; ‡‡*p* < 0.01 for comparison of Δ*yqiC* in aerobic culture vs. anaerobic culture)

To investigate the effect of *yqiC* on the five complexes of the ETC, a qRT-PCR was performed to examine the mRNA expression of the five representative genes; the results revealed the significant downregulation of *nuoE*, *ndh*, *sdhB*, and *cydA* in Δ*yqiC* and the significant upregulation of *cyoC* after the depletion of *yqiC* in *S*. Typhimurium SL1344 in aerobic culture (Fig. 9A) and anaerobic culture (Fig. 9B). To further study the effect of oxygen on the aforementioned effect, the two aforementioned sets of mRNA expression data were further compared. The comparison revealed that the downregulation trends of *nuoE*, *ndh*, *sdhB*, and *cydA* in Δ*yqiC* relative to those of SL1344 were similar in both anaerobic and aerobic cultures; however, a nonsignificant difference in lower fold changes was detected. Notably, relative to the mRNA expression of *cyoC* in SL1344, that of *cyoC* in Δ*yqiC* was more significantly upregulated in anaerobic culture than in aerobic culture (2.17 ± 0.12 vs. 1.5 ± 0.1 fold change, *p* = 0.005). In general, the restoration of *yqiC* in Δ*yqiC*' partially reversed the effects of *yqiC* regulation on the aforementioned genes such that their mRNA expression levels approached those detected in SL1344 (Fig. 9). An RNA-seq analysis was conducted to compare the ETC-associated genes in Δ*yqiC* with those in *S*. Typhimurium SL1344 after 2 h of in vitro infection in Caco-2 cells: the analysis revealed that the depletion of *yqiC* significantly upregulated *ndh*, *sdhA*, and *appB* and caused upregulation trends for *nuoA*, *sdhB*, and *cyoA*; however, no significant regulation of *nuoE*, *cyoC*, or *cydA* was detected (Additional file 9: Table S9), indicating that a reverse regulation occurred after the interaction of *S*. Typhimurium with Caco-2 cells.

### *yqiC* is required for maintaining NADH/NAD^+^ redox status and H_2_O_2_ production

**Fig. 10:**
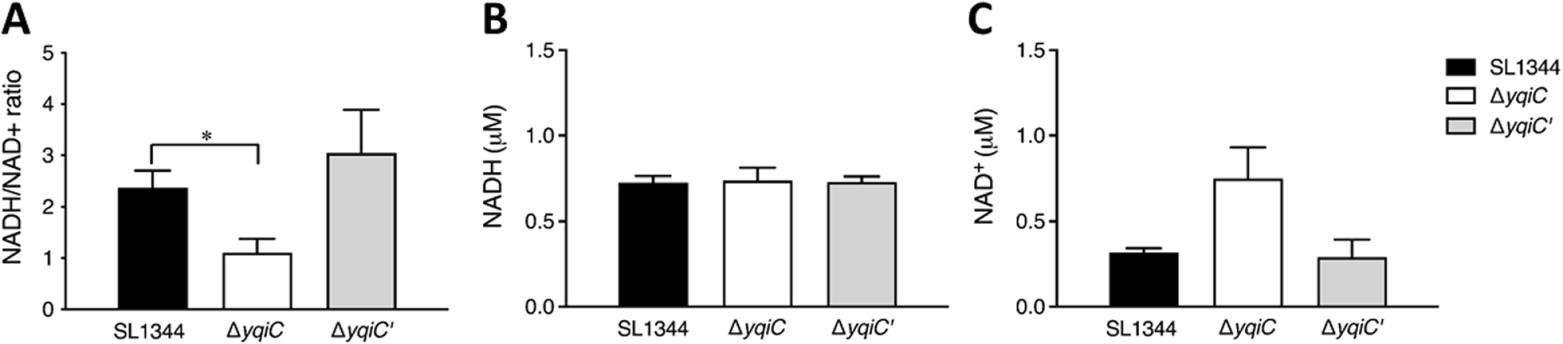
NADH/NAD^+^ ratios (**A**), NADH concentrations (**B**), and NAD^+^ concentrations (**C**) as obtained from mid-log cultures of *S*. Typhimurium SL1344, Δ*yqiC*, and Δ*yqiC'* through independent experiments performed in triplicate; NADH/NAD^+^ ratios are significantly decreased in *S*. Typhimurium Δ*yqiC* relative to its paternal wild-type SL1344, which is mainly attributed to increases in NAD^+^ concentrations while NADH concentrations remain unchanged (*p < 0.05)

**Fig. 11:**
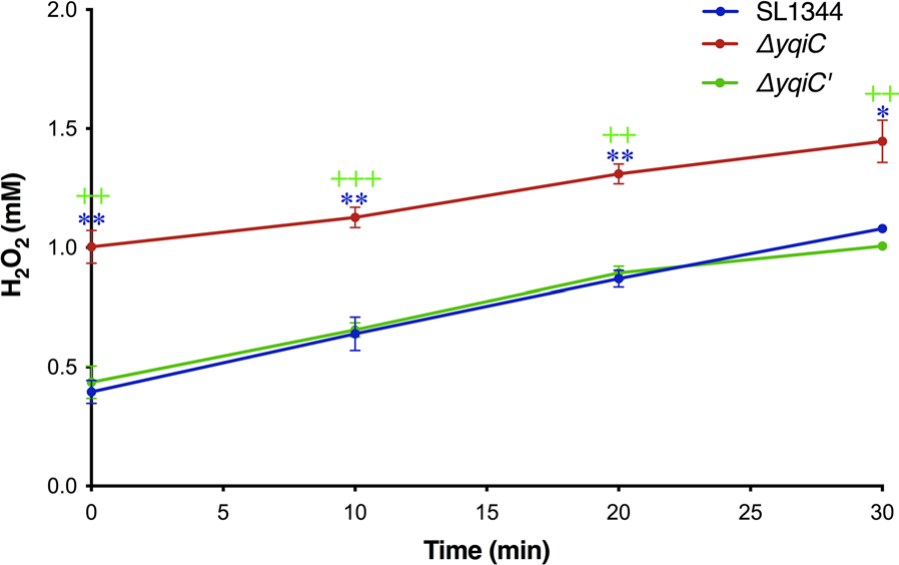
H_2_O_2_ assays performed independently in triplicate. H_2_O_2_ concentrations in *S*. Typhimurium Δ*yqiC* are significantly higher than those in its paternal wild-type SL1344 and *yqiC*-complemented strain Δ*yqiC*'. *p < 0.05 and **p < 0.01 for comparisons between Δ*yqiC* and SL1344; ^++^*p* < 0.01 and ^+++^*p* < 0.001 for comparisons between Δ*yqiC* and Δ*yqiC*'

Our NADH/NAD^+^ assays indicated that the NADH/NAD^+^ ratios of Δ*yqiC* were significantly decreased relative to those of SL1344 (Fig. 10A). This decrease in NADH/NAD^+^ ratios due to the depletion of *yqiC* occurred mainly because the NADH concentration in Δ*yqiC* was unchanged (Fig. 10B) but its NAD^+^ concentration significantly increased (Fig. 10C). Additionally, our H_2_O_2_ assays revealed that the H_2_O_2_ concentrations in Δ*yqiC* were significantly higher than those in SL1344 and Δ*yqiC*' (Fig. 11).

## Discussion

The current RNA-seq study clarified the effects of *yqiC* during the colonization of Caco-2 cells on the expression of other *Salmonella* genes, particularly the negative regulation that occurs during pyrimidine and spermidine biosynthesis, osmoprotection, and DNA transcription and the positive regulation that occurs in *ilvB* operon, the *tdc* family, anaerobic dimethylsulfoxide reductase, the cytochrome c family, and NADH dehydrogenase. A few studies have reported the association of the aforementioned genes with bacterial colonization but not in *ilvL* (or *ilvB* operon) and *dms* genes. Early studies of *E.* *coli* and *S*. Typhimurium have revealed that *carAB*, *pyrBI*, *pyrC*, *pyrD*, *pyrE*, and *pyrF* are required for the biosynthesis of uridine monophosphate, which is the precursor of all pyrimidine nucleotides. The expression of *pyr* operons is repressed by nucleotides through the transcription attenuation control mechanism [27]. The *pyrE*–deleted mutant exhibited a defect in the intestinal colonization of *S*. Typhimurium in chicks that cannot be restored by the salvage pathway, indicating the necessity of *pyrE* and de novo pyrimidine synthesis for colonization [28]. Our RNA-seq results indicate *yqiC* inhibits the upregulation of *pyrB*, *pyrI*, *pyrE*, *pyrD*, and *pyrC* during *S*. Typhimurium colonization, and our GO analysis results indicate the involvement of *pyrB* in the cellular amino acid metabolic process and involvement of *pyrI* in metal ion binding. In addition, polyamines are essential for biofilm formation in *E.* *coli*. PotFGHI functions as a compensatory importer of spermidine when PotABCD is absent under biofilm-forming conditions [27]. In the present study, *potF* was identified in 117 significantly upregulated genes and in the periplasmic space (through a GO analysis) after the depletion of *yqiC* in *S*. Typhimurium; this finding is consistent with the subcellular localization of YqiC [3]. *S*. Typhimurium in chicken intestine lumen significantly upregulates the expression of the *potFGHI* operon [29]. Therefore, *potF* can be negatively regulated by *yqiC* to affect *Salmonella* colonization. Moreover, the transient activation of *tdcA* in *S*. Typhimurium when bacterial growth shifted from aerobic to anaerobic growth; a *tdcA* mutation reduced the expression of the genes involved in flagellar biosynthesis, downregulated the expression of *tdcBCDEG*, and induced the expression of genes associated with energy metabolism, suggesting activation of carbon catabolism genes for cellular energy production before the full synthesis of ATP from anaerobic ETCs [30]. In addition, our GO analysis of the *tdc* family revealed the involvement of *tdcA* in DNA-binding transcription factor activity and DNA-templated transcription, the involvement of *tdcB* in pyridoxal phosphate binding, and the involvement of *tdcD* in metal ion binding. The association of *nrdD* and *nrfA* with colonization was reported for other bacteria of the *Enterobacteriaceae* family than *Salmonella*. The knockout of *nrdD* attenuated the colonization of an adherent-invasive *E.* *coli* strain in murine gut mucosa [31], and a *nrfA*-disrupted mutant of *Campylobacter jejuni* significantly attenuated colonization in chicks [32]. Our GO analysis revealed the involvement of *nrfA* in the five clusters, namely iron ion binding, heme binding, periplasmic space, microbial metabolism in diverse environments, and nitrogen metabolism.

The present study validated the findings of our previous study related to the characterization of non-SPI gene *yqiC* with respect to its role in regulating type 1 fimbriae, SPI-1 genes, and flagellin in *S*. Typihmurium SL1344 [4], which is similar to the phenotype of a SPI-19 locus SEN1005 in *S*. Enteritidis [33]. The *invH*-mediated Sip effector proteins are important in early cecal inflammation by *S*. Typhimurium in mice colitis [34]. Accordingly, *sipA*, *sipC*, and *sipD* were identified in the nine SPI-1 significantly downregulated genes of our RNA-seq analysis, emapplot analysis, and KEGG analysis. However, the role of *yqiC* in modulating SPI-2 genes is complex. Mutation in the SPI-2 gene *hha* induces no defect in *S*. Typhimurium colonization to the host gut [35], and this gene is not influenced by *yqiC* in Caco-2 cells by our RNA-seq analysis. In contrast to our previous finding regarding the downregulation of one representative SPI-2 gene *sseB* in Δ*yqiC*, the present RNA-seq revealed the diverse regulation of SPI-2 genes, including the downregulation of *spvB*, *ttrS*, and *ttrA* and upregulation of *sseE* and *sscA*; these findings suggest the presence of a complex mechanism involving the bidirectional regulation of *yqiC* and SPI-2 genes. In addition, the associations of SPI-3, SPI-4, SPI-5, and SPI-6 with bacterial colonization or with the intestinal lumen have been sporadically reported. The nonmotile and nonchemotactic *S*. Typhimurium in chicken intestinal lumen has been reported to exhibit the upregulation of SPI-3 (*mgtC*, *rmbA*, *fidL*, *shdA,* and *misL*) and SPI-5 (*pipB*) genes, suggesting a close physical interaction with the host during colonization [29]. The SPI-3 gene-encoded MisL and the SPI-4 gene-encoded SiiC, SiiD, and SiiF assemble T1SS to secrete SiiE for the adhesion of *Salmonella* to intestinal epithelial cells during gut colonization [36, 37]. Similarly, a study of global transcriptomes revealed that the SPI-4 genes (*siiABCDEF*), the SPI-5 genes (*sopB*, *pipB*, and *sigE*), and the SPI-6 genes (*sciJKNOR*) are responsible for the colonization of *S.* *enterica* serovar Dublin in bovine mammary epithelial cells [38]. The knockout of *yqiC* significantly downregulated the *ydiA* that encodes conserved hypothetical plasmid protein, suggesting that *yqiC* is required for expressing SPI-3 *ydiA*. Similar to the effect of cell association on SPI-4 and SPI-5 gene expression [38], colonization-associated *yqiC* significantly downregulated the SPI-4 gene *siiD* and the SPI-5 gene *pipC*. Although the SPI-6 genes *sciJKNOR* were downregulated in *S.* *enterica* serovar Dublin, we discovered that other SPI-6 genes *safA* and *sciC* were downregulated in *S*. Typhimurium after the depletion of *yqiC* and loss of colonization ability. Overall, the expression of type-1 fimbriae, SPI-1, and flagellin were regulated by *yqiC*, and several genes of SPI-2, -3, -4, -5, and -6 interacted with *yqiC* through unknown mechanisms that require further investigation.

To our knowledge, the biosynthesis of UQ-8 in *E*. *coli* requires the enzymes encoded by at least 15 *ubi* genes, including *ubiC*, *ubiA*, *ubiD/X*, *ubiI*, *ubiB*, *ubiH*, *ubiE*, *ubiF*, *ubiG*, *ubiH*, *ubiJ*, *ubiK* [7, 12, 39], *ubiT*, *ubiU*, and *ubiV* [40] through a novel oxygen-independent pathway. The biosynthesis of MK-8 in *E*. *coli* requires at least nine *men* genes, namely, *menF*, *menD*, *menH*, *menC*, *menE*, *menB*, *menI*, *menA*, and *menG* (also referred to as *ubiE*). The main difference between UQ and MK biosynthesis is that chorismate is converted into 4-hydroxybenzoate through UbiC for UQ synthesis and into isochorismate through MenF for MK synthesis [12, 41]. However, UQ and MK biosynthesis are not fully separate pathways. Required for the biosynthesis of both UQ and MK, UbiE (MenG), which is encoded by *ubiE*, is a nonspecific enzyme that can catalyze the C-methylation of 2-octaprenyl-6-methoxy-1,4-benzoquinol into 2-octaprenyl-3-methyl-6-methoxy-1,4-benzoquinol in UQ biosynthesis; it also catalyzes the methylation of DMK-8 to MK-8 in the final step of MK biosynthesis in *E.* *coli* [42]. In the *E.* *coli* strain MG1655 (alignment of *yqiC* is 77% identical to that of *S*. Typhimurium SL1344), the *ubiI* mutant had the highest correlation with the *yqiC* (also named *ubiK*) mutant that reduced UQ-8 to 18% and slightly increased MK-8 under aerobic conditions but was not detected under anaerobic conditions. In the *S* *enterica* strain 12,023 (alignment of *yqiC* is 75% identical to that of *S*. Typhimurium SL1344), the *ubiK* mutant also caused a 16-fold decrease in UQ-8, but no significant difference in MK-8 level was detected in the WT strain under aerobic conditions [7]. However, our previous study reported that the absence of MK in *S*. Typhimurium SL1344 after the depletion of *yqiC* and the addition of MK reversed the effect of *yqiC* depletion on the expression of type-1 fimbrial, flagellar, SPI-1, and SPI-2 genes, which indicated the significant influence of MK on *yqiC* and its role as a upstream regulator of the virulence and ETC of *S*. Typhimurium [4]. In the present study, *yqiC* was required for expressing *menD*. It requires more studies for validating whether *yqiC* serves as a regulator in the ETC through the modulation of *men* and/or *ubi* genes for maintaining the homeostasis between UQ and MK biosynthesis under various circumstances.

Studies have indicated the involvement of molybdenum, iron, and sulfur in bacterial virulence. Molybdoprotein oxidoreductase is an iron–sulfur cluster that is homologous to *phs* operon, which encodes thiosulfate reductase for thiosulfate reduction to contribute to the anaerobic energy metabolism in *S*. Typihmurium [43]. The phylogenetic tree of the molybdenum subunits that form the dimethyl sulfoxide reductase superfamily in *E* *coli* includes *ttr*, *dms*, *nar*, and *nap* genes [44], which were present in our main emapplot network of Δ*yqiC*, indicating the close relationship of *yqiC* with molybdenum and iron–sulfur subunits. NarG is the only nitrate reductase for the colonization of *E* *coli* in mouse intestines [45]. Under acute tolerance response, lacking of *narZ* encoding the nitrate reductase subunit NarZ results in *S*. Typhimurium deficiency and upregulation of *dsrA* encoding sRNA DsrA are associated with motility, adhesion, and invasion efficacy [46, 47], which were not found in our *yqiC* study in Caco-2 cells. However, *napA* disruption significantly attenuated the colonization by *C*. *jejuni* in the cecum of chickens [32]. The *napA* mutant of *S*. Typhimurium exhibited a considerable growth defect in the low-nitrate colonic lumen of mice [48]; by contrast, the highest mortality rates of chickens challenged with mutants of *S.* gallinarum were associated with mutations in *napA* and *narG*, and additional attenuations were induced by a mutation in *frdA* and double mutations in *dmsA* and *torC* [49]. The findings are consistent with our emapplot network of downregulation in *napA*, *dmsA*, *torC*, and *narG* after the mutation in *yqiC* that is associated with colonization and ETCs. In particular, during the early colonization of *S*. Typhimurium with Caco-2 cells, *yqiC* was required for expressing the six genes that encode dimethylsulfoxide reductase subunit A (*napA*), dimethylsulfoxide reductase subunit A (*dmsA2*, *dmsA1*, and *dmsA*), and two other unnamed genes (Fig. 3B). In addition, we discovered that *yqiC* downregulates *ttrA* to affect tetrathionate dehydrogenase. These four enzymes contribute to bacterial virulence and belong to the dimethyl sulfoxide reductase family; they have a subcellular location and exhibit a common structure comprising a Mo-containing subunit, an iron–sulfur protein, and a membrane-bound subunit with or without binding hemes [50].

The mechanisms involved cellular respiration in *Salmonella* virulence associated with bacterial colonization in hosts remain unclear. A cluster genes related to the carbohydrate metabolism and transportation required for intestinal colonization was identified using a library of targeted single-gene deletion mutants of *S*. Typhimurium inoculated in the ligated ileal loops of calves [51], and *S*. Typhimurium was revealed to use carbohydrates and their metabolites through the phosphoenolpyruvate-dependent phosphotransferase system [52]. A study compared the global transcriptomes of highly pathogenic *S.* *enterica* serovar Dublin and the less pathogenic *S.* *enterica* serovar Cerro in their interactions with bovine mammary epithelial cells and identified the *S.* *enterica* genes responsible for *Salmonella* infection and colonization in cattle, including the genes associated with carbohydrate transport/metabolism, energy production/metabolism, and coenzyme transport/metabolism [38]. A proteomic study of *S*. Typhimurium during the infection of HeLa epithelial cells revealed the preferential use of glycolysis, the pentose phosphate pathway, mixed acid fermentation, and nucleotide metabolism and the repression of the TCA cycle and aerobic and anaerobic respiration pathways [53]. *S*. Typhimurium performs an incomplete TCA cycle in the anaerobic mammalian gut; however, a complete oxidative TCA cycle can be induced by inflammation-derived electron acceptors such that microbiota-derived succinate can be used as a carbon source during intestinal colonization [54]. These findings are also reflected in our discovery of the decreased ATP production in *S*. Typhimurium after the deletion of *yqiC*, which is attributed to the complex role of *yqiC* in influencing the contribution of glycolysis, TCA cycles, and ETCs to the cell respiration that converts these nutrients into ATP.

The Seahorse XFp Analyzer was used to measure glycolysis and mitochondria respiration in the mammalian cells [55], and eukaryotic cells, including *Caenorhabditis elegans* (nematode) [56], *Dictyostelium discoideum* (amoeba) [57], *Candida albicans* [58], and *Cryptococcus neoformans* [59]. Cellular respiration plays a similar role in mitochondrial respiration, and several studies have used the Seahorse XFp Analyzer to investigate mitochondria-absent prokaryotic bacteria such as *E.* *coli*, *Staphylococcus aureus* [24, 60] and *Mycobacterium tuberculosis* [61]; however, in this context, the research on *Salmonella* is limited. In the Seahorse Analyzer, ampicillin at a dose of 5 × MIC or 50 × MIC accelerates cellular respiration by increasing OCR, indicating the association of antibiotic efficacy and phenotypic resistance with cellular respiration in *E.* *coli* [24, 60]. By contrast, in our study, sublethal ampicillin did not have a considerable effect on the OCR and ECFR of *S*. Typhimurium or the phenotype of *yqiC*. A study that used the Seahorse Analyzer revealed that the iron–sulfur cluster biosynthesis protein SufT is required for glycolysis, oxidative phosphorylation, and survival in *Mycobacterium tuberculosis* after exposure to oxidative stress and nitric oxide [61]; this finding echoes our findings regarding the association of *yqiC* with electron transfer activity, iron–sulfur cluster assembly, and glycolysis in *S*. Typhimurium. Our RNA-seq analysis revealed the involvement of *yqiC* in energy and carbohydrate metabolism, and a series of experiments in the Seahorse XFp Analyzer further clarified how *yqiC* influences cellular respiration and glycolysis. Our cell phenotype energy test verified that *yqiC* influences cellular respiration more than glycolysis to maintain metabolic potential, which is achieved by inhibiting ATP synthase and uncoupling oxidative phosphorylation; this finding suggests that other metabolic pathways are responsible for increased oxygen consumption under energy stress. Furthermore, we revealed that *yqiC* is required for sufficient glycolysis and the maintenance of glycolytic capacity and glycolytic reserve. Therefore, the colonization-associated gene *yqiC* is expected to assist NTS in acquiring energy through cellular respiration and glycolysis to express NTS virulence, and oxygen consumption plays a major role in cellular respiration under energy stress conditions. Collectively, these findings are consistent with our previous findings regarding the phenotyping of *yqiC* (*ubiK*) as a regulator for the efficient aerobic biosynthesis of UQ and MK [4, 7]; however, the involved anaerobic effect requires further clarification.

*S*. Typhimurium and *E.* *coli* may differ with respect to the regulation of ETC complexes. The mutations in *nuo* and *cyd* operons suppressed the anaerobic growth of *S*. Typhimurium [15]. In addition, mutations in the *nuoG*, *nuoM*, and *nuoN* of NDH-1 not only rescue motility, growth, and the rate of aerobic respiration but also use L-malate as the sole carbon source in a *S*. Typhimurium *ubiA*–*ubiE* mutant, suggesting that *nuoG*, *nuoM*, and *nuoN* suppress the electron flow activity of NDH-1 [14]. Both *ubiA* and *ubiE* mutations do not lead to UQ biosynthesis and reduce the quinone pool, in which only *ubiA* mutations cause higher biosynthesis of MK than of DMK and only *ubiE* mutations deter the biosynthesis of UQ and MK while DMK biosynthesis continues to occur in *S*. Typhimurium [14]; this finding suggests that these *nuo* genes are negative regulators that influence the bridging roles of *ubiA* and *ubiE*, and *ubiE* in maintaining the equilibrium among UQ, MK, and DMK compositions in the total quinone pool. Researchers have explored the relationships of ETC complex genes with NTS growth. The *S*. gallinarum *nuoG* mutant was reported to be highly attenuated in the colonization that occurred in the caeca of chickens and the invasions that occurred in the liver or spleen of chickens [62]. The *S*. Typhimurium genes involved in energy production and conversion (i.e., *nuoJ*, *nuoI*, *napC*, *cyoD*, *frdD*, *nuoE*, *nuoF*, *cyoC*, and *cydA*) were downregulated during colonization in chicken cecal lumen relative to their expression in broth cultures [29]. By contrast, we examined the effects of *yqiC* on the expression of the five selected genes of the electron donating complexes NDH-1 (Nuo) and NDH-2 (Ndh), succinate dehydrogenase (SDH), and the electron accepting complexes cytochrome bo oxidases (Cyo) and cytochrome bd oxidases (Cyd) in the ETC of *S*. Typhimurium [13, 29] and the anaerobic effect on their expression. Our analysis indicated that *yqiC* depletion downregulated the expression of *nuoE*, *ndh*, *sdhB*, and *cydA* in both aerobic and anaerobic *S*. Typhimurium. However, *yqiC* depletion significantly upregulated the *cyoC* expression that was further reinforced by anaerobiosis, suggesting that *yqiC* is a suppressor of the expression of *cyoC* for receiving electrons in the ETC, particularly in anaerobic *S*. Typhimurium. This effect of *yqiC* depletion on the downregulation of *nuoE*, *ndh*, *sdhB*, and *cydA* and the upregulation of *cyoC* was reversed by *S*. Typhimurium colonization in Caco-2 cells with the significant upregulation of *ndh* and *sdhB*. The distinctive phenotype of the *cyo* genes from other ETC genes was also revealed in a study to exhibit *cyo* gene–involved cytochrome bo oxidase but not cytochrome bd-I and bd-II oxidases; therefore, it significantly contributes to the release of extracellular ATP in *E* *coli* and *Salmonella* and the survival of bacterial communities, playing roles in bacterial physiology other than that of an energy supplier [63]. The exposure of *S* Typhimurium to anaerobiosis enhances virulence, adhesion to enterocytes and the penetration of mucus into host cells [64]. Therefore, the modulation of *yqiC* in ETC complexes changes from downregulation to upregulation during colonization, and the unique expression of *cyoC* may play a role in the virulence of *S*. Typhimurium during its early interaction with intestinal epithelium.

The NADH/NAD^+^ ratio is a key metabolic marker of cellular state for balance in bacterial redox and for environmental adaptability, and a change in this ratio can influence metabolite distribution through the involvement of carbon sources under various oxidative states [25, 65]. Under aerobic conditions, *E.* *coli* uses the respiratory chain to oxidize NADH to NAD^+^ and channels redox energy to generate a proton gradient for ATP synthase. Anaerobically grown *E.* *coli* regenerates NAD^+^ from intermediates (e.g., pyruvate, oxaloacetic acids, malate, and acetyl-CoA) with NADH when no other electron acceptors (e.g., nitrate) are present [25, 65]. The NADH/NAD^+^ ratio is moderately adjusted by various carbon sources; the *E.* *coli* that is aerobically grown on acetate is an exception because it exhibits a considerably higher NADH/NAD^+^ ratio than that of glucose [25]. In addition to the TCA cycle, the *S*. Typhimurium within epithelial cells can generate acetate and lactate under aerobic conditions through the overflow metabolism with the simultaneous synthesis of ATP and NADH [16]. The total NADH/NAD^+^ intracellular pool is maintained in *E.* *coli* by NAD biosynthesis through the de novo pathway and by NAD recycling through the pyridine nucleotide salvage pathway. NAD does not limit metabolic rates because the generation of NADH (conversion of formate to CO_2_ and H_2_) and regeneration of NAD^+^ (efflux of succinate, ethanol, and lactate) can redistribute the metabolic fluxes in the central anaerobic metabolic pathway [66]. At present, the contribution of ETC complexes to NADH/NAD^+^ metabolism in bacteria is poorly understood. NADH/NAD^+^ ratios increased when mutations occurred in two genes (*nuo F* and *ndh*) encoding NADH dehydrogenase and three genes (*cydB*, *cyoB*, and *appB*) encoding cytochrome oxidases in aerobic *E.* *coli* [25], indicating that the expression of these genes is responsible for the maintenance of a stabilized NADH/NAD^+^ ratio and that these enzymes can convert NADH to NAD^+^ in an ETC or either increase NADH or reduce NAD^+^ in other pathways (e.g., conversion of formate into CO_2_ [66], glycolysis, and the TCA cycle [16, 25, 65]). In addition, the NADH/NAD + ratios of aerobic *E.* *coli* are only approximately half of those of anaerobic *E.* *coli* [25], suggesting that NADH is a greater contributor than NAD^+^ to anaerobiosis.

In *E*. *coli*, the electron transfer in the respiratory chain blocked by bactericidal peptidoglycan recognition proteins (PRGPs) can suppress the NADH oxidoreductases NDH-1 and NDH-2, increase the NADH/NAD^+^ ratio after the supply of NADH from glycolysis and the TCA cycle is increased, divert electrons from NADH oxidoreductases to O_2_, and generate H_2_O_2_ to increase oxidative stress that kills bacteria [22]. The diversion of electrons flow from formate dehydrogenase FDH-O, NDH-1, and NDH-2, and cytochrome bd-I with incomplete electron transfer from UQ-H2 or its malfunction can serve as another ETC component that enables the excessive production of H_2_O_2_ from O_2_ to induce oxidative stress [67]. We demonstrated that in *S*. Typhimurium, *yqiC* is required for expressing *nuoE*, *ndh*, *sdhB*, and *cydA* in the ETC of aerobic and anaerobic grown *S*. Typhimurium and for expressing *fdhF* encoding formate dehydrogenase during the colonization in Caco-2 cells (Additional file 9: Table S9). However, the colonization in Caco-2 cells reversed the *yqiC* regulation in *nuo*, *ndh*, *sdhB*, and *cyd* and caused their expression to be repressed, suggesting the key role of *yqiC* in modulating ROS through these ETC components before and during colonization. We discovered that the repression of *cyoC* expression in *cyoC* in aerobic and anaerobic grown *S*. Typhimurium was stronger under anaerobic conditions than under aerobic conditions; however, this regulation was reduced by colonization (Additional file 9: Table S9). H_2_O_2_ is an ROS that is generated by oxidative stress inside the *Salmonella*-containing vacuoles that exist within phagocytes or exist intrinsically in bacteria because of the respiratory chain or indirect action of antibiotics [68]. Most studies of ROS in *S*. Typhimurium have reported the ability of intracellular bacteria to survive in macrophages or neutrophils; however, few studies have studied ROS in bacteria that interact with intestinal epithelial cells. The deletion of the *arcA* of aerobic grown *S*. Typhimurium in vitro led to increased ROS production and an increased NADH/NAD^+^ ratio [69]. In neutrophils and macrophages, *S*. Typhimurium *arcA* downregulates *ompD* and *ompF* in the presence of H_2_O_2_ in vitro [70]. H_2_O_2_ stress increases the mRNA expression levels of porin-encoding *ompX* but not those of proteins, indicating the complex posttranscriptional regulation of *ompX* under oxidative stress [71]. In the present study, *yqiC* had no effect on *arcABC* genes, but *yqiC* was required for expressing *ompN*, *ompS*, and *ompW* but not *ompX* and other *omp* genes after the infection of Caco-2 cells with *S*. Typhimurium (Additional file 10: Table S10). Moreover, ROS production can be bactericidal in host or can be used by *S*. Typhimurium to induce virulence genes for colonization [72]. To induce virulence genes for colonization, inflammation-associated ROS production can generate tetrathionate as a respiratory electron pool through *S*. Typhimurium in an anaerobic environment (e.g., the gut) [73]. In anaerobic respiration, *S.* *enterica* can be differentiated from *E.* *coli* by its use of tetrathionate and thiosulfate as electron acceptors for tetrathionate reduction and sulfide formation [11]. In our study through RNA-seq analysis, emapplot of GO enrichment analysis, and KEGG pathways, *yqiC* was required for expressing *ttrA* and *ttrS* encoding SPI-2 T3SS effectors, and it is also involved in molybdopterin cofactor binding, iron–sulfur cluster binding, and metal ion binding (*ttrA*), sulfur metabolism and microbial metabolism in diverse environments (*ttrC* and *ttrA*), and the two-component system (*ttrC*, *ttrA*, and *ttrS*) after the priming of *S*. Typhimurium with Caco-2 cells. Collectively, our previous study revealed that *yqiC* and NADH dehydrogenase inhibitor rotenone are similar in terms of their effect on the expression of flagella and repression of type-1 fimbria [4]; it also indicated that *yqiC* is associated with ETC components and subsequent intrinsic ROS production such that it plays a key role in balancing oxidative stress and bacterial pathogenicity in *S*. Typhimurium.

The present study has several limitations. First, not all of the results obtained through the RNA-seq analysis were validated through qRT-PCR. Second, the knockout of specific genes of interest was not performed in *S*. Typhimurium. However, for the present study, a strict criterion for statistical significance was set, and the 20 most significantly regulated genes as identified through the RNA-seq analysis were validated.

## Conclusions

In this study, a list of unreported genes highly regulated by the colonization-associated gene *yqiC* in NTS were identified for the first time, and the key roles and possible mechanisms of *yqiC* in virulence factors, SPIs, UQ and MK biosynthesis, ETCs, glycolysis, and oxidative stress were revealed. Because *yqiC* is essential for the successful early colonization of NTS in host cells, how *yqiC* manipulates the aforementioned modules and whether its pathways involved in early colonization could be blocked by specific molecules can provide a resolution of combating NTS infection. The present study provides useful insights that further the understanding of the *yqiC*-involved signaling pathways and regulatory network, which should be further studied to clarify and develop new therapeutic strategies against NTS.

## Supplementary Information

**Additional file 1: Table S1.** Sequences of primers used for qRT-PCR for mRNA expression of the ten most significantly upregulated genes and downregulated genes (as identified by comparative RNA-seq analysis of Δ*yqiC* and *S*. Typhimurim SL1344), and the housekeeping 16S ribosomal RNA gene.

**Additional file 2: Table S2.** Sequences of primers used for qRT-PCR for determining the mRNA expression of genes representing five complexes of electron transport chain in *Salmonella* and housekeeping 16S ribosomal RNA gene.

**Additional file 3: Table S3.** RNA-seq analysis showing the 117 most significantly upregulated genes (A) and the 291 most significantly downregulated genes (B) of Δ*yqiC* relative to *S*. Typhimurium SL1344 after in vitro infection with Caco-2 cells for 2 h.

**Additional file 4: Table S4.** Summary of the genes in the 30 cluster groups obtained from emapplot of GO enrichment analysis for RNA-seq of Δ*yqiC* relative to *S*. Typhimurium SL1344.

**Additional file 5: Table S5.** RNA-seq analysis for the genes encoding T3SS structures, effectors, or regulation proteins of SPI-1, SPI-2, SPI-3, SPI-4, SPI-5, and SPI-6 of Δ*yqiC* relative to *S*. Typhimurium SL1344 after in vitro infection with Caco-2 cells for 2 h.

**Additional file 6: Table S6.** RNA-seq analysis for the fifteen genes involved in ubiquinone biosynthesis (A) and the nine genes associated with menaquinone biosynthesis (B) of Δ*yqiC* relative to *S*. Typhimurium SL1344 after in vitro infection with Caco-2 cells for 2 h.

**Additional file 7: Table S7.** Detailed gene information in 30 clusters as obtained from an emapplot of a GO enrichment analysis.

**Additional file 8: Table S8.** Genes in 19 KEGG pathways as identified through RNA-seq analysis of Δ*yqiC* relative to *S*. Typhimurium SL1344 after in vitro infection with Caco-2 cells for 2 h.

**Additional file 9: Table S9.** RNA-seq analysis for the ETC and cytochrome-associated genes (*nuo*, *ndh*, *sdhB*, *cydA*, and *fdhF*) of Δ*yqiC* relative to *S*. Typhimurium SL1344 after in vitro infection with Caco-2 cells for 2 h.

**Additional file 10: Table S10.** RNA-seq analysis for the *omp* genes of Δ*yqiC* relative to *S*. Typhimurium SL1344 after in vitro infection with Caco-2 cells for 2 h.

**Additional file 11: Fig. S1.** KEGG pathway of RNA-seq for *yqiC* involvement in sulfur metabolism.

**Additional file 12: Fig. S2.** KEGG pathway of RNA-seq indicating *yqiC*–regulated genes in *Salmonella* infection.

**Additional file 13: Fig. S3.** KEGG pathway of RNA-seq indicating significantly downregulated genes involved in bacterial invasion of epithelial cells after *yqiC* deletion during *Salmonella* infection.

**Additional file 14: Fig. S4.** KEGG pathway of RNA-seq indicating significantly downregulated genes involved in glycolysis/gluconeogenesis after *yqiC* deletion in *S*. Typhimurium.

**Additional file 15: Fig. S5.** KEGG pathway of RNA-seq indicating pathways involved in carbon metabolism after *yqiC* deletion in *S*. Typhimurium.

**Additional file 16: Fig. S6.** KEGG pathway of RNA-seq indicating involvement of *yqiC* in microbial metabolism in diverse environments after *yqiC* deletion in *S*. Typhimurium.

**Additional file 17: Fig. S7.** KEGG pathway of RNA-seq indicating involvement of *yqiC* in ascorbate and aldarate metabolism after *yqiC* deletion in *S*. Typhimurium.

**Additional file 18: Fig. S8.** KEGG pathway of RNA-seq indicating involvement of *yqiC* in biosynthesis of secondary metabolite after *yqiC* deletion in *S*. Typhimurium.

**Additional file 19: Fig. S9.** KEGG pathway of RNA-seq indicating involvement of *yqiC* in nitrogen metabolism after *yqiC* deletion in *S*. Typhimurium.

## Data Availability

All data generated or analysed during this study are included in this published article and its additional information files.

## Abbreviations

NTS: Nontyphoidal *Salmonella*
SPI: *Salmonella* pathogenicity island
BMFP: Brucella membrane fusogenic protein
UQ: Ubiquinone
ROS: Reactive oxygen species
ATP: Adenosine triphosphate
TCA: Tricarboxylatic acid
ETC: Electron transport chain
MK: Menaquinone
DMK: Demethylmenaquinone
*S*. Typhimurium: *Salmonella enterica* subsp. *enterica* serovar Typhimurium
GO: Gene Ontology
KEGG: Kyoto Encyclopedia of Genes and Genomes
OCR: Oxygen consumption rate
ECAR: Extracellular acidification rate
MIC: Minimum inhibition concentration
FCCP: Carbonyl cyanide 4-(trifluoromethoxy) phenylhydrazone
2-DG: 2-Deoxyglucose
