# Supplementary material for: Effects of colonization-associated gene yqiC on global transcriptome, cellular respiration, and oxidative stress in Salmonella Typhimurium
Source: J Biomed Sci. 2022 Dec 1;29:102. doi: 10.1186/s12929-022-00885-0 (PMC9714038; doi:10.1186/s12929-022-00885-0)
Supplement: Supplementary file 1 — Additional file 1: Table S1. Sequences of primers used for qRT-PCR for mRNA expression of the ten most significantly upregulated genes and downregulated genes (as identified by comparative RNA-seq analysis of ΔyqiC and S. Typhimurim SL1344), and the housekeeping 16S ribosomal RNA gene. [file 12929_2022_885_MOESM1_ESM.docx]

Table S1. Sequences of primers used for qRT-PCR for mRNA expression of the ten most significantly upregulated genes and downregulated genes (as identified by comparative RNA-seq analysis of Δ*yqiC* and *S*. Typhimurim SL1344), and the housekeeping 16S ribosomal RNA gene.

| Gene/target ID | Sequence (5′ to 3′)  (F: forward, R: reverse) | Product size (base pairs) | Description |
| --- | --- | --- | --- |
| *pyrB* | F: TCATTTCCATAAACGACCTCA  R: TGTCGGAGAAGCCCACTA | 201 | Aspartate carbamoyltransferase catalytic subunit |
| *pyrI* | F: GCACCGTGATTGACCATA  R: GGGCAAACCAGCACATTA | 286 | Aspartate carbamoyltransferase regulatory subunit |
| *carA* | F: TGTCGGCACCAATAAAGC  R: TTTCTCACGCAGCAGTCG | 190 | Carbamoyl phosphate synthase small subunit |
| *osmX* | F: CCTTCAGCAAGCCTACCA  R: AGCACCTCTTTACGCACC | 216 | Glycine/betaine ABC transporter substrate-binding protein |
| *carB* | F: GTTAGGGCCAGAAATGCG  R: TGATACGGTCCTGAATGTGC | 299 | Carbamoyl phosphate synthase large subunit |
| *fimH* | F: ATCCCTCGCCAGACAATG  R: GCCGAAATCAAACTCCAC | 153 | Fimbrial adhesin FimH |
| *fimA* | F: CTATTGCGAGTCTGATGTTTG  R: CAGGACGATGGAGAAAGG | 224 | Type 1 fimbrial protein subunit A |
| *hydA* | F: ACGGCGTCTCCTTTCCTA  R: CCCTGGGTTTGATACTGGTTA | 297 | [Ni/Fe] hydrogenase small subunit |
| *fimI* | F: TTCGTTACGGTTAATCTCCTG  R: CGCTGCTGGTCATCAAATA | 159 | Fimbrial protein subunit FimI |
| SL1344_RS06225 | F: GCTACAGGACCATCACCG  R: ATACCCAATACCGTTTGC | 119 | Hypothetical protein |
| *ivbL* | F: GAACGCGACCCTACTAACAAC  R: TTGCCGACGACCACCACCAC | 72 | *ilvB* operon leader peptide IvbL |
| *yqiC* | F: ACCAGTTCAGGGAAACCA  R: GCTGAGATTGCAGCGTTT | 141 | Hypothetical protein |
| *ybaM* | F: AACTGGCCGTGGATTTAA  R: GCGAGTCGTCTTCCGTAC | 126 | DUF2496 domain-containing protein |
| *cbtA* | F: CTACGTTTGTCGATGCCG  R: CTTTCCTGTCGTGTCCGT | 132 | Hypothetical protein |
| SL1344_RS08230 | F: TAATGAAATAATTCGCAGTG  R: CTGGAAAGCAAAGGTTAA | 175 | Hypothetical protein |
| *tdcB* | F: TGCTCCACGGTGACAACT  R: GCAATACCTGCAATTAAACC | 205 | Threonine ammonia-lyase |
| *tdcA* | F: CTTTCCTTCGCTGATTGG  R: CATGTTCGGGACTTGCTG | 243 | Transcriptional regulator TdcA |
| *fljB* | F: ACGGTACAGTAACCCTTGC  R: CGTAATCTGCGGCGTAAT | 264 | Flagellin |
| *fljA* | F: GATTATTCTATGCTTGCTCGTC  R: CAATGCGCTTATTTCCTTT | 166 | Phase 1 flagellin transcriptional repressor |
| *argF* | F: CGAAGATGGGCATGGATA  R: CAGGCTTCTTTGGGTTCTC | 199 | Putative ornithine carbamoyltransferase |
| 16S rRNA | F: TTCCTCCAGATCTCTACGCA  R:GTGGCTAATACCGCATAACG | 552 | Housekeeping gene 16S ribosomal RNA gene |
