# Supplementary material for: Effects of colonization-associated gene yqiC on global transcriptome, cellular respiration, and oxidative stress in Salmonella Typhimurium
Source: J Biomed Sci. 2022 Dec 1;29:102. doi: 10.1186/s12929-022-00885-0 (PMC9714038; doi:10.1186/s12929-022-00885-0)
Supplement: Supplementary file 2 — Additional file 2: Table S2. Sequences of primers used for qRT-PCR for determining the mRNA expression of genes representing five complexes of electron transport chain in Salmonella and housekeeping 16S ribosomal RNA gene. [file 12929_2022_885_MOESM2_ESM.docx]

Table S2. Sequences of primers used for qRT-PCR for determining the mRNA expression of genes representing five ETC components in *S*. Typhimurium and the housekeeping 16s ribosomal RNA gene.

| Gene | Sequence (5′ to 3′)  (F: forward, R: reverse) | Product size (base pairs) | Description |
| --- | --- | --- | --- |
| *nuoE* | F: TCATTTCCATAAACGACCTCA  R: CCCGGTTTGATGTTGAGATT | 186 | Complex I NADH: ubiquinone oxidoreductase I (NDH-1) |
| *ndh* | F: AATGGCGACGCAGTTAGG  R: TCGGCAATGGTGATGGTT | 237 | Complex I NADH: ubiquinone oxidoreductase II (NDH-2) |
| *sdhB* | F: TCCCAGCCTTTCTTTCCG  R: GCGGGCGAATCACAATCT | 143 | Complex II |
| *cyoC* | F: GGCTGGCGTTGACCTTCT  R: TGCGGGTACGGTTAGTGC | 228 | Cytochrome bo terminal oxidase |
| *cydA* | F: GCTGCCGTACTGTCCGTTAT  R: GGTTGCGTTTCCCATTCA | 107 | Cytochrome bd-oxidases |
| 16S rRNA | F: TTCCTCCAGATCTCTACGCA  R:GTGGCTAATACCGCATAACG | 552 | Housekeeping gene 16S ribosomal RNA gene |
