# Supplementary material for: Effects of colonization-associated gene yqiC on global transcriptome, cellular respiration, and oxidative stress in Salmonella Typhimurium
Source: J Biomed Sci. 2022 Dec 1;29:102. doi: 10.1186/s12929-022-00885-0 (PMC9714038; doi:10.1186/s12929-022-00885-0)
Supplement: Supplementary file 4 — Additional file 4: Table S4. Summary of the genes in the 30 cluster groups obtained from emapplot of GO enrichment analysis for RNA-seq of ΔyqiC relative to S. Typhimurium SL1344. [file 12929_2022_885_MOESM4_ESM.docx]

| **No.** | **ID** | **Description** | **Gene ratio** | **Bg ratio** | **Adjusted *p* value** | ***q* value** | **Upregulated genes** | **Downregulated genes** | **Count** |
| --- | --- | --- | --- | --- | --- | --- | --- | --- | --- |
| 1 | GO:0006189 | De novo IMP biosynthetic process | 8/522 | 12/5258 | 0.000365 | 0.000303 | *purT*, *purC*, *purM*, *purG*, *purD*, *purH*, *purK*, *purE* | – | 8 |
| 2 | GO:0043546 | Molybdopterin cofactor binding | 8/522 | 15/5258 | 0.001808 | 0.0015 | – | *ttrA*, SL1344_RS07445, SL1344_RS07450, *narG*, *napA*, SL1344_RS12970, SL1344_RS22105, *dmsA* | 8 |
| 3 | GO:0005506 | Iron ion binding | 13/522 | 39/5258 | 0.002206 | 0.00183 | *yfhF*, *nifU*, *yfhP*, *yhgI* | *fhuE*, *cybH*, *napA*, *yhgG*, *torC*, *nrfA*, *fepA*, *fes* | 12 |
| 4 | GO:0030151 | Molybdenum ion binding | 6/522 | 10/5258 | 0.004069 | 0.003375 |  | SL1344_RS07445, SL1344_RS07450, *napA*, SL1344_RS12970, SL1344_RS22105, *dmsA* | 6 |
| 5 | GO:0016226 | Iron–sulfur cluster assembly | 6/522 | 11/5258 | 0.006562 | 0.005444 | *hscA*, *yfhF*, *nifU*, *yhgI* | *sufB*, *sufD* | 6 |
| 6 | GO:0009055 | Electron transfer activity | 15/522 | 62/5258 | 0.016199 | 0.013439 | SL1344_RS06220, *fdx*, *sdhC* | SL1344_RS07445, SL1344_RS07450, *cybH*, *narG*, *cybH*, *napA*, SL1344_RS12970, SL1344_RS19665, *torC*, SL1344_RS22105, *fixA*, *dmsA* | 15 |
| 7 | GO:0051536 | Iron–sulfur cluster binding | 7/522 | 18/5258 | 0.018458 | 0.015313 | *yfhF*, *nifU* | *sufA*, *ttrA*, *glpC*, *yhcC*, *yhgG* | 7 |
| 8 | GO:0003700 | DNA binding transcription factor activity | 27/522 | 164/5258 | 0.071916 | 0.059662 | *putA*, *pdhR*, SL1344_RS11200, *yfhP*, *rpoS*, *metR*, *yijO*, *adiY* | *yebK*, *fliA*, *pocR*, SL1344_RS11710, *fljA*, SL1344_RS14515, *srlR*, SL1344_RS15070, SL1344_RS15110, *tdcA*, *malT*, SL1344_RS19485, SL1344_RS19740, *melR*, SL1344_RS22705, SL1344_RS22895, SL1344_RS02845, *dpiA*, SL1344_RS03865 | 27 |
| 9 | GO:0051539 | 4 iron, 4 sulfur cluster binding | 15/522 | 75/5258 | 0.071916 | 0.059662 | SL1344_RS08915, *yhgI*, *fdoH* | SL1344_RS07445, SL1344_RS07450, *narG*, *napA*, SL1344_RS12970, *hypO*, SL1344_RS00185, SL1344_RS22105, *nrdG*, *yjjW*, *moaA*, *dmsA* | 15 |
| 10 | GO:0009103 | Lipopolysaccharide biosynthetic process | 6/522 | 18/5258 | 0.071916 | 0.059662 | *yeiU*, *yfbE*, *pmrF*, *yfbG*, *pqaB* | *wzc* | 6 |
| 11 | GO:0016301 | Kinase activity | 10/522 | 44/5258 | 0.101573 | 0.084267 | *pykF*, *prsA* | SL1344_RS06580, *pyk*, *wzc*, *fruF*, SL1344_RS12025, *lyxK*, SL1344_RS19480, *nagE* | 10 |
| 12 | GO:0009289 | Pilus | 7/522 | 26/5258 | 0.110365 | 0.091561 | *fimA*, *fimI*, *fimH*, *fimF* | *stcA*, *stdA*, *sthD* | 7 |
| 13 | GO:0020037 | Heme binding | 6/522 | 21/5258 | 0.125073 | 0.103763 | – | *napC*, SL1344_RS19665, *torC*, *nrfA*, *nrfB*, *nrfE* | 6 |
| 14 | GO:0030170 | Pyridoxal phosphate binding | 9/522 | 41/5258 | 0.141313 | 0.117236 | *nifS*, *glyA*, *lysA*, *ybdL* | *sufS*, *yfbQ*, SL1344_RS14515, *tdcB*, *cobD* | 9 |
| 15 | GO:0006777 | Mo-molybdopterin cofactor biosynthetic process | 4/522 | 11/5258 | 0.141313 | 0.117236 |  | *napA*, *mobA*, *moaA*, *moaE* | 4 |
| 16 | GO:0006351 | Transcription, DNA-templated | 28/522 | 190/5258 | 0.150522 | 0.124876 | *rcsA*, SL1344_RS11200, SL1344_RS18725, *metR*, *yijO*, *adiY*, *glnK*, *fimZ*, *fimW* | *yebK*, *sdiA*, *pocR*, SL1344_RS11710, SL1344_RS12115, SL1344_RS14515, *srlR*, SL1344_RS15070, *tdcA*, *malT*, SL1344_RS19485, SL1344_RS19740, *melR*, SL1344_RS22705, *idnR*, SL1344_RS23265, SL1344_RS02845, *caiF*, SL1344_RS03865 | 28 |
| 17 | GO:0042597 | Periplasmic space | 9/522 | 43/5258 | 0.15272 | 0.126699 | SL1344_RS07380, SL1344_RS07780, *ais*, *cpxP*, *potF* | *napA*, SL1344_RS16505, *nrfA*, *nrfB* | 9 |
| 18 | GO:0005576 | Extracellular region | 4/522 | 12/5258 | 0.15272 | 0.126699 | – | *sopE*, *fljB*, *sipA*, *sipC* | 4 |
| 19 | GO:0009236 | Cobalamin biosynthetic process | 6/522 | 24/5258 | 0.15272 | 0.126699 | *purC* | *cbiT*, *cbiD*, *cibB*, *cbiA*, *cobD* | 6 |
| 20 | GO:0006520 | Cellular amino acid metabolic process | 5/522 | 18/5258 | 0.15272 | 0.126699 | *speC*, *pyrB* | *eutC*, *cadA*, *speF* | 5 |
| 21 | GO:0009245 | Lipid A biosynthetic process | 5/522 | 18/5258 | 0.15272 | 0.126699 | *yeiU*, *yfbE*, *pmrF*, *yfbG*, *pqaB* | – | 5 |
| 22 | GO:0007155 | Cell adhesion | 7/522 | 31/5258 | 0.156097 | 0.1295 | *fimA*, *fimI*, *fimH*, *fimF* | *stcA*, *stdA*, *sthD* | 7 |
| 23 | GO:0006541 | Glutamine metabolic process | 4/522 | 13/5258 | 0.170216 | 0.141214 | *purG*, *carA* | *cbiA*, *asnB* | 4 |
| 24 | GO:0009401 | Phosphoenolpyruvate-dependent sugar phosphotransferase system | 11/522 | 62/5258 | 0.194152 | 0.161072 | – | *yadI*, *fruA*, *fruF*, SL1344_RS12015, *srlE*, *frwC*, *sgaT*, SL1344_RS23265, SL1344_RS23280, SL1344_RS23285, *nagE* | 11 |
| 25 | GO:0046872 | Metal ion binding | 32/522 | 240/5258 | 0.232117 | 0.192568 | *aceE*, SL1344_RS08915, *hybD*, *guaB*, *nifS*, *purG*, *queD*, *accC*, *fdoH*, *purD*, *pyrI*, *purK*, *carB*, *sdhC* | *ttrA*, *narG*, *napC*, *eutG*, *gutQ*, *hypO*, *tdcD*, SL1344_RS00185, SL1344_RS19665, *mobA*, *fucO*, *pfkA*, *gldA*, *nrfB*, *nrfE*, *cpdB*, *nagA*, *moaA* | 32 |
| 26 | GO:0046677 | Response to antibiotics | 3/522 | 10/5258 | 0.31212 | 0.25894 | *yfbE*, *pmrF*, *yfbG* |  | 3 |
| 27 | GO:0009405 | Pathogenesis | 6/522 | 31/5258 | 0.334303 | 0.277343 | – | *sigE*, *sopE*, *sipA*, *sipD*, *sipC*, *sopD* | 6 |
| 28 | GO:0008483 | Transaminase activity | 3/522 | 11/5258 | 0.334303 | 0.277343 | *ybdL* | *yfbQ*, *cobD* | 3 |
| 29 | GO:0009297 | Pilus assembly | 3/522 | 11/5258 | 0.334303 | 0.277343 | *fimD* | *stcC*, *stdB* | 3 |
| 30 | GO:0015473 | Fimbrial usher porin activity | 3/522 | 11/5258 | 0.334303 | 0.277343 | *fimD* | *stcC*, *stdB* | 3 |
