# Supplementary material for: Effects of colonization-associated gene yqiC on global transcriptome, cellular respiration, and oxidative stress in Salmonella Typhimurium
Source: J Biomed Sci. 2022 Dec 1;29:102. doi: 10.1186/s12929-022-00885-0 (PMC9714038; doi:10.1186/s12929-022-00885-0)
Supplement: Supplementary file 8 — Additional file 8: Table S8. Genes in 19 KEGG pathways as identified through RNA-seq analysis of ΔyqiC relative to S. Typhimurium SL1344 after in vitro infection with Caco-2 cells for 2 h. [file 12929_2022_885_MOESM8_ESM.docx]

| **No.** | **Pathway ID** | **Description** | **Gene Ratio** | **Bg Ratio** | ***p*. adjust** | ***q* value** | **Upregulated genes** | **Downregulated genes** | **Count** |
| --- | --- | --- | --- | --- | --- | --- | --- | --- | --- |
| 1 | [sey00920](file:///C:\Users\Shiuh-Bin\AppData\Roaming\Microsoft\Excel\KEGG_KK_Yqic_vs_SL1344_FC2X\sey00920.welgene.png) | Sulfur metabolism | 14/189 | 38/1724 | 0.00138 | 0.00117 | *glpE* | SL1344_RS22110, SL1344_RS22105, SL1344_RS12970, *dmsC*, *dmsB*, *cysJ*, *sbp*, SL1344_RS07440, *ttrC*, *ttrA* | 14 |
| 2 | [sey05100](file:///C:\Users\Shiuh-Bin\AppData\Roaming\Microsoft\Excel\KEGG_KK_Yqic_vs_SL1344_FC2X\sey05100.welgene.png) | Bacterial invasion of epithelial cells | 6/189 | 10/1724 | 0.00788 | 0.00671 | – | *sopD*, *sopE*, *sipD*, *sipC*, *sptP*, *sipA* | 6 |
| 3 | [sey01120](file:///C:\Users\Shiuh-Bin\AppData\Roaming\Microsoft\Excel\KEGG_KK_Yqic_vs_SL1344_FC2X\sey01120.welgene.png) | Microbial metabolism in diverse environments | 46/189 | 267/1724 | 0.01128 | 0.0096 | *hyaA*, *prs*, *lysA*, *metF*, *glpE*, *sdhC*, SL1344_RS08920, *aceE*, *sdhA*, *fdoH*, *glyA*, *aceF*, *pykF*, *accC* | SL1344_RS12015, SL1344_RS22910, SL1344_RS12025,  *eutG*, *nrfA*, *hpaC*, SL1344_RS12010, *hypO*, *fucA*, *mgsA*, *cysJ*, *hpaB*, *ttrC*, *ttrA*, *sgaT*, *iolD*, *hxlA*, *fruB*, *tdcE*, *yihR*, SL1344_RS11185,  *eutE*, *ydiF*, *narH*, SL1344_RS20785,  *pykA*, *napA*, *narG*, *edd*, *hybC*, *pfkA*, *fruA* | 46 |
| 4 | [sey01130](file:///C:\Users\Shiuh-Bin\AppData\Roaming\Microsoft\Excel\KEGG_KK_Yqic_vs_SL1344_FC2X\sey01130.welgene.png) | Biosynthesis of antibiotics | 39/189 | 223/1724 | 0.01861 | 0.01584 | *aroQ*, *purK*, *purT*, *purH*, *prs*, *purC*, *lysA*, *putA*, *sdhC*, *purD*, *purM*, *purG*, *aceE*, *sdhA*, *purE*, *speC*, *glyA*, *aceF*, *pykF*, *accC* | *tdcB*, SL1344_RS22905, SL1344_RS22915, *speF*, *entA*, *entB*, *entD*, *eutG*, *entF*, SL1344_RS12010, *ilvC*, *entE*, *yihR*, *nagA*, *entC*, SL1344_RS20785, *pykA*, *pfkA*, *yfbQ* | 39 |
| 5 | [sey00250](file:///C:\Users\Shiuh-Bin\AppData\Roaming\Microsoft\Excel\KEGG_KK_Yqic_vs_SL1344_FC2X\sey00250.welgene.png) | Alanine, aspartate and glutamate metabolism | 10/189 | 32/1724 | 0.01996 | 0.017 | *pyrB*, *pyrI*, *carA*, *carB*, *putA* | *asnB*, *asnA*, *aspA*, *ansB*, *yfbQ* | 10 |
| 6 | [sey01110](file:///C:\Users\Shiuh-Bin\AppData\Roaming\Microsoft\Excel\KEGG_KK_Yqic_vs_SL1344_FC2X\sey01110.welgene.png) | Biosynthesis of secondary metabolites | 50/189 | 314/1724 | 0.02102 | 0.0179 | *aroQ*, *purK*, *purT*, *purH*, *prs*, *purC*, *lysA*, *glpE*, *guaB*, *putA*, *sdhC*, *purD*, *purM*, *purG*, *aceE*, *sdhA*, *purE*, *speC*, *glyA*, *aceF*, *pykF*, *accC* | *tdcB*, SL1344_RS22905, SL1344_RS22915, *cadA*, *speF*, *asnB*, *glpC*, *asnA*, *entA*, *entB*, *entD*, *glpA*, *eutG*, *entF*, *flhAb*, SL1344_RS12010, *menD*, *ilvC*, *entE*, *leuD2*, *yihR*, *ansB*, *entC*, SL1344_RS20785, *wrbA*, *pykA*, *pfkA*, *yfbQ* | 50 |
| 7 | [sey00230](file:///C:\Users\Shiuh-Bin\AppData\Roaming\Microsoft\Excel\KEGG_KK_Yqic_vs_SL1344_FC2X\sey00230.welgene.png) | Purine metabolism | 17/189 | 83/1724 | 0.06449 | 0.05491 | *purK*, *purT*, *purH*, *prs*, *purC*, *guaB*, *purD*, *purM*, *purG*, *purE*, *pykF* | SL1344_RS22910, *flhAb*, *cpdB*, *nrdE*, *nrdD*, *pykA* | 17 |
| 8 | [sey05132](file:///C:\Users\Shiuh-Bin\AppData\Roaming\Microsoft\Excel\KEGG_KK_Yqic_vs_SL1344_FC2X\sey05132.welgene.png) | *Salmonella* infection | 8/189 | 28/1724 | 0.06855 | 0.05836 | – | *fljB*, *nrfA*, *sopE*, *sipD*, *sipC*, *spvB*, *sptP*, *sipA* | 8 |
| 9 | [sey00640](file:///C:\Users\Shiuh-Bin\AppData\Roaming\Microsoft\Excel\KEGG_KK_Yqic_vs_SL1344_FC2X\sey00640.welgene.png) | Propanoate metabolism | 9/189 | 40/1724 | 0.1902 | 0.16194 | *accC* | *tdcD*, *mgsA*, *prpB*, *tdcE*, *prpD*, *ydiF*, *prpC*, *gldA* | 9 |
| 10 | [sey00053](file:///C:\Users\Shiuh-Bin\AppData\Roaming\Microsoft\Excel\KEGG_KK_Yqic_vs_SL1344_FC2X\sey00053.welgene.png) | Ascorbate and aldarate metabolism | 6/189 | 23/1724 | 0.2204 | 0.18765 | *udg* | SL1344_RS12015, SL1344_RS12025, *lyxK*, *sgaT*, *garD* | 6 |
| 11 | [sey02020](file:///C:\Users\Shiuh-Bin\AppData\Roaming\Microsoft\Excel\KEGG_KK_Yqic_vs_SL1344_FC2X\sey02020.welgene.png) | Two-component system | 27/189 | 176/1724 | 0.2204 | 0.18765 | *hyaC*, *fimZ*, *yfbE*, *pstS*, *rcsA*, *appB*, *basS* | *fljB*, *fepA*, *citF*, *ttrC*, *ttrA*, *kdpB*, *sdiA*, *torC*, *tsr*, *citB*, *citA*, *hyaC2*, *narH*, *tcp*, SL1344_RS11875, *flgM*, *narQ*, *fliA*, *narG*, *ttrS* | 27 |
| 12 | [sey00010](file:///C:\Users\Shiuh-Bin\AppData\Roaming\Microsoft\Excel\KEGG_KK_Yqic_vs_SL1344_FC2X\sey00010.welgene.png) | Glycolysis / Gluconeogenesis | 9/189 | 43/1724 | 0.2204 | 0.18765 | *aceE*, *aceF*, *pykF* | *eutG*, SL1344_RS19435, *yihR*, SL1344_RS20785, *pykA*, *pfkA* | 9 |
| 13 | [sey00670](file:///C:\Users\Shiuh-Bin\AppData\Roaming\Microsoft\Excel\KEGG_KK_Yqic_vs_SL1344_FC2X\sey00670.welgene.png) | One carbon pool by folate | 4/189 | 13/1724 | 0.23654 | 0.20139 | *purT*, *purH*, *metF*, *glyA* |  | 4 |
| 14 | [sey02060](file:///C:\Users\Shiuh-Bin\AppData\Roaming\Microsoft\Excel\KEGG_KK_Yqic_vs_SL1344_FC2X\sey02060.welgene.png) | Phosphotransferase system (PTS) | 10/189 | 57/1724 | 0.41301 | 0.35164 | – | SL1344_RS12015, SL1344_RS12025, *sgaT*, SL1344_RS19480, *fruB*, *srlE*, SL1344_RS23280, *nagE*, SL1344_RS23285, *fruA* | 10 |
| 15 | [sey00240](file:///C:\Users\Shiuh-Bin\AppData\Roaming\Microsoft\Excel\KEGG_KK_Yqic_vs_SL1344_FC2X\sey00240.welgene.png) | Pyrimidine metabolism | 10/189 | 58/1724 | 0.41301 | 0.35164 | *pyrB*, *pyrI*, *carA*, *carB*, *pyrE*, *pyrD*, *pyrC* | *cpdB*, *nrdE*, *nrdD* | 10 |
| 16 | [sey00910](file:///C:\Users\Shiuh-Bin\AppData\Roaming\Microsoft\Excel\KEGG_KK_Yqic_vs_SL1344_FC2X\sey00910.welgene.png) | Nitrogen metabolism | 5/189 | 23/1724 | 0.41301 | 0.35164 | – | SL1344_RS22910, *nrfA*, *narH*, *napA*, *narG* | 5 |
| 17 | [sey01200](file:///C:\Users\Shiuh-Bin\AppData\Roaming\Microsoft\Excel\KEGG_KK_Yqic_vs_SL1344_FC2X\sey01200.welgene.png) | Carbon metabolism | 17/189 | 113/1724 | 0.41301 | 0.35164 | *prs*, *metF*, *sdhC*, *aceE*, *sdhA*, *fdoH*, *glyA*, *aceF*, *pykF*, *accC* | *tdcB*, SL1344_RS22910, SL1344_RS12010, *hxlA*, *pykA*, *edd*, *pfkA* | 17 |
| 18 | [sey00790](file:///C:\Users\Shiuh-Bin\AppData\Roaming\Microsoft\Excel\KEGG_KK_Yqic_vs_SL1344_FC2X\sey00790.welgene.png) | Folate biosynthesis | 5/189 | 24/1724 | 0.42965 | 0.3658 | *ptpS* | *moaA*, SL1344_RS15260, *mobA*, *moaE* | 5 |
| 19 | [sey00220](file:///C:\Users\Shiuh-Bin\AppData\Roaming\Microsoft\Excel\KEGG_KK_Yqic_vs_SL1344_FC2X\sey00220.welgene.png) | Arginine biosynthesis | 4/189 | 18/1724 | 0.45069 | 0.38372 | – | SL1344_RS22905, SL1344_RS22915, SL1344_RS22910, *yfbQ* | 4 |
